# Communicative signals during joint attention promote neural processes of infants and caregivers

**DOI:** 10.1016/j.dcn.2023.101321

**Published:** 2023-12-06

**Authors:** Anna Bánki, Moritz Köster, Radoslaw Martin Cichy, Stefanie Hoehl

**Affiliations:** a University of Vienna, Faculty of Psychology, Vienna, Austria; b University of Regensburg, Institute for Psychology, Regensburg, Germany; c Freie Universität Berlin, Faculty of Education and Psychology, Berlin, Germany

**Keywords:** Hyperscanning, Infancy, Joint attention, Ostensive cues, Steady-state visually evoked potentials, Visual perception

## Abstract

Communicative signals such as eye contact increase infants’ brain activation to visual stimuli and promote joint attention. Our study assessed whether communicative signals during joint attention enhance infant-caregiver dyads’ neural responses to objects, and their neural synchrony. To track mutual attention processes, we applied rhythmic visual stimulation (RVS), presenting images of objects to 12-month-old infants and their mothers (n = 37 dyads), while we recorded dyads’ brain activity (i.e., steady-state visual evoked potentials, SSVEPs) with electroencephalography (EEG) hyperscanning. Within dyads, mothers either communicatively showed the images to their infant or watched the images without communicative engagement. Communicative cues increased infants’ and mothers’ SSVEPs at central-occipital-parietal, and central electrode sites, respectively. Infants showed significantly more gaze behaviour to images during communicative engagement. Dyadic neural synchrony (SSVEP amplitude envelope correlations, AECs) was not modulated by communicative cues. Taken together, maternal communicative cues in joint attention increase infants’ neural responses to objects, and shape mothers’ own attention processes. We show that communicative cues enhance cortical visual processing, thus play an essential role in social learning. Future studies need to elucidate the effect of communicative cues on neural synchrony during joint attention. Finally, our study introduces RVS to study infant-caregiver neural dynamics in social contexts.

## Highlights

• Communicative cues enhance brain activity of infants and caregivers.
• Eye contact, pointing and infant-directed-speech shape infants’ attention.
• Rhythmic visual stimulation aids the study of joint attention.
• No evidence of higher neural synchrony due to ostensive cues in present task.

## 1 Introduction

Infants share early visual experiences with their caregivers when engaging in joint attention, one of the most important building blocks of social-cognitive development and social competence (Mundy and Newell, 2007). During joint attention, infants and caregivers coordinate their attention between self, others, and objects in the environment to adopt a common point of reference and focus on socially relevant information (Bakeman and Adamson, 1984, Siposova and Carpenter, 2019). Infants begin to respond to joint attention from the age of 2–3 months and start to initiate joint attention through mutual engagement by the age of 8–9 months (Butterworth, 2007, Carpenter et al., 1998, Mundy, 2018, Mundy et al., 2007). Mutual engagement in joint attention is achieved via communicative cues, other-awareness, and early forms of perspective-taking (Csibra and Gergely, 2006, Moll and Meltzoff, 2011, Reddy, 2018, Siposova and Carpenter, 2019, Tomasello, 1995, Tomasello and Moll, 2010). Communicative signals such as eye contact, pointing, and infant-directed speech (IDS) convey communicative intent, structure infants’ attention in social interactions (Okumura et al., 2020, Senju and Csibra, 2008), and facilitate social learning (Csibra and Gergely, 2006, Csibra and Gergely, 2009, Wass et al., 2018a, Wass et al., 2020b). The current study aimed to examine the effects of communicative signals on infant-caregiver dyads’ brain activity to provide new insights into the interpersonal neural dynamics of joint attention during early social interactions.

The social coordination of attention between infants and adults such as caregivers has been extensively studied on the behavioural and neural level in controlled experimental settings (Bakeman and Adamson, 1984, Grossmann et al., 2008, Hoehl and Bertenthal, 2021, Mundy, 2018, Mundy and Newell, 2007, Salley and Colombo, 2016, Siposova and Carpenter, 2019, Tomasello, 1995), with a particular focus on communicative signals that have been shown to facilitate object learning in the first postnatal year (Çetinçelik et al., 2021, Cleveland and Striano, 2007, Michel et al., 2019, Reid and Striano, 2005, Striano et al., 2006a, Sun and Yoshida, 2022, Thiele et al., 2021, Wass et al., 2018b, Yu et al., 2019, Yu and Smith, 2013, Yu and Smith, 2016). Prior studies demonstrated that infants’ neural processing of objects is facilitated by eye contact: At 5 and 9 months of age, infants’ attention is enhanced when looking at objects following direct eye contact vs no eye contact with an adult, marked by a higher negative component (Nc) of the ERP (Parise et al., 2008, Striano et al., 2006b). Infants at 9 months show a desynchronization of alpha-band activity reflecting cortical excitation when looking at objects following mutual eye contact with an adult (Hoehl et al., 2014). These findings highlight the importance of communicative cues in modulating brain activity, even though the above studies applied structured experimental tasks and did not involve free-flowing naturalistic interactions.

Other communicative signals such as IDS and pointing also enhance infants’ attention and facilitate learning (Carpenter et al., 1998, Daum et al., 2013, Liszkowski, 2018, Nencheva and Lew-Williams, 2022, Parise and Csibra, 2013, Suarez-Rivera et al., 2019, Yu and Smith, 2012). Even though primarily studied in a non-interactionist context, IDS and pointing have been found to elicit distinct neural responses in infants: When listening to IDS vs backward human speech, 6-month-old infants showed event-related desynchronisation to IDS in the 4–9 Hz EEG frequency range as a sign of higher attention allocation (Woodruff Carr et al., 2021). Since IDS has less predictable speech contours than adult-directed speech (ADS), it leads to attention increases and in turn, better learning outcomes (Räsänen et al., 2018). However, in another study with 7-month-olds, IDS, and ADS similarly enhanced infants’ attention-specific neural responses to audio-visual displays (Peykarjou et al., 2020). Regarding pointing, 8-month-olds’ neural responses (P400 of the ERP) were increased by congruent vs incongruent pointing gestures to target objects displayed on a screen (Gredebäck et al., 2010).

Together, these results indicate that communicative cues in joint attention with a social partner increase infants’ attentiveness and facilitate learning, including object processing (Hoehl et al., 2012; Okumura et al., 2016; Reid et al., 2004; Siposova and Carpenter, 2019) and later recognition (Kopp and Lindenberger, 2011) in structured experimental settings. Yet, most early social interactions unfold in naturalistic, dyadic contexts directly between infants and their caregivers (Hoehl and Markova, 2018, Markova et al., 2019, Wass et al., 2020b). Thus, recent theoretical advances in the field called for a more interactionist and dynamic approach to studying infants’ social attention abilities, for instance, by assessing fine-tuned mutual adjustments in the infant-caregiver dyad during joint attention (Hoehl and Bertenthal, 2021, Phillips and Wass, 2021, Wass and Goupil, 2022). According to the second-person approach in social-interactional neuroscience, behaviour and brain activity are remarkably different when one engages in a live social interaction in contrast to passively observing social stimuli (Hoehl and Markova, 2018, Redcay and Schilbach, 2019, Schilbach et al., 2013). Hence, recording behavioral and neural dynamics simultaneously from infants and their caregivers during a real joint attention interaction (i.e., with hyperscanning) can offer invaluable insights into the evolving attentional dynamics within the dyad. This approach will allow for tracking the attention allocation of infant and caregiver simultaneously, as well as the adult-led modulations of infant attention, bringing important advancement to the field. We argue that studying the neural underpinnings of joint attention through hyperscanning offers a deeper understanding of the attentional processes involved beyond individual brain measures alone. Extending our study to caregivers’ behaviour and brain activity, instead of focusing solely on the infant, and implementing more naturalistic experimental settings will lead to a comprehensive understanding of how infants learn in social interactions.

While most prior research focused on the neural underpinnings of joint attention and its development in infants (Eggebrecht et al., 2017, Elison et al., 2013, Grossmann and Johnson, 2010, Mundy et al., 2000, Mundy and Jarrold, 2010, Rayson et al., 2019, Smith et al., 2021), here we investigated how joint attention and maternal communicative cues shape the neural processes of mother-infant dyads and contribute to neural synchrony in a close-to-naturalistic social interaction. Neural synchrony is characterized by temporally co-occurring patterns of brain activity between two individuals (Wass et al., 2020b) that is considered a core mechanism to support information transfer via verbal and non-verbal communication during social interactions (Hasson et al., 2012). Positive infant-caregiver interactions are characterized by higher neural synchrony during communication (e.g., Endevelt-Shapira and Feldman, 2023; Piazza et al., 2020) and social bonding (e.g., Nguyen et al., 2021). Given that neural synchrony likely reflects socially aligned dynamic attention processes (Dikker et al., 2017), it is reasonable to assume that it could be implicated in joint attention and early social learning. Wass et al. (2020b) put forward that communicative cues can lead to a concurrent phase-reset of neural oscillations of infant and caregiver during social interaction, ensuring high neural excitability for more efficient information encoding. In addition, prior studies with adults found that joint attention accompanied with eye contact modulated individuals’ brain activity (e.g., Lachat et al., 2012) or interpersonal neural synchrony within the dyad during a naturalistic social interaction (e.g., Dravida et al., 2020; Koul et al., 2023; Luft et al., 2022). Based on these findings, we propose that communicative cues accompanying joint attention will elicit a higher degree of attention alignment between infant and caregiver, which will be reflected in more similar neural activity in the dyad, and thus higher neural synchrony in EEG measurements.

Recent studies that explored infant-caregiver neural dynamics in naturalistic joint attention contexts found predictive links between infant-caregiver dyads’ attention, gaze behaviour and neural activity (Phillips et al., 2023, Wass et al., 2018b, Wass et al., 2020a). During joint free play, twelve-month-old infants’ attention changes were tracked by their caregiver's neural responsivity (i.e., increased EEG theta power) that facilitated infants’ sustained attention to objects (Wass et al., 2018b) and may drive neural synchrony (Wass et al., 2020a). Importantly, the episodes when infants were more attentive also led to increased parental neural activity in the theta oscillatory band. The authors suggest that this down-shifting from alpha to the theta oscillatory band in caregivers serves to track infants’ attention dynamics mainly characterized by theta oscillations. The study also found that higher parental neural responsiveness made infants more attentive during social play. This shows that caregivers display neural responsivity to the infant’s behaviour, and this increased responsivity is associated with infant attentiveness. A subsequent study with 12-month-olds demonstrated that even though infants' neural activity did not increase before infant- vs adult-led joint attention episodes, it was sensitive to the caregiver joining the infant’s attention, indicating anticipatory processing (i.e., increased alpha suppression) (Phillips et al., 2023). These findings indicate that infant-caregiver dyads display neural tracking of each other’s attention and behaviour during joint attention interactions, suggesting that exploring mutual neural dynamics and neural synchrony in the dyad in these instances could be highly informative. However, relatively few studies assessed the direct effect of joint attention on infant-caregiver neural synchrony during live, reciprocal social interactions (Wass et al., 2020b, Wass and Goupil, 2022). While former studies reported increased neural synchrony associated with mutual eye contact between infants and adults (Leong et al., 2017, Leong et al., 2019), in a recent study with 12-month-old infants engaging in free play with a caregiver, mutual gaze onsets did not seem to be associated with changes in neural synchrony (Marriott Haresign et al., 2023).

It is important to note that studies reporting infant-caregiver dyads’ EEG activity in naturalistic contexts face several challenges. These include EEG data being highly prone to eye and movement artifacts (Georgieva et al., 2020; Hoehl & Wahl, 2012; Marriott Haresign et al., 2022) and the difficulty in interpreting neural phase synchrony measures between identical but functionally different neural oscillatory bands in infant and adult EEG (Nguyen et al., 2020, Saby and Marshall, 2012). Therefore, several studies have employed functional near-infrared spectroscopy (fNIRS) rather than dual-EEG to study neural interactional dynamics between infants and their caregivers (e.g., Nguyen et al., 2021, Nguyen et al., 2023). For instance, one study showed that 9–15-month-olds’ neural synchrony with an adult preceded and anticipated mutual gaze during natural communication and free play (Piazza et al., 2020). Yet, the temporal resolution of fNIRS is limited as it captures hemodynamic processes, thus brain dynamics in much lower frequency bands. An outstanding task is to develop new methods to investigate caregiver-infant neural dynamics in naturalistic interactions with temporally fine-grained yet robust assessments of brain activity. The current study addresses this challenge by applying a close-to-naturalistic paradigm that allows to explore the inter-dyadic attentional dynamics of shared attention between infant and caregiver.

Here, we assess how communicative signals such as eye contact, IDS, and pointing simultaneously affect the brain activity of 11–12-month-old infants and their caregivers engaging in a naturalistic but controlled joint attention interaction, specifically during sustained attention to objects displayed on a screen. Brain activity is characterized by rhythmic oscillations indicating dynamic fluctuations in the excitability of neurons. Here, we applied the method of rhythmic visual stimulation (RVS, i.e., presenting flickering visual stimuli during EEG recording) to entrain infant-caregiver dyads’ neuronal oscillations, leading to increased activity specifically in the stimulated frequency; so-called steady-state visually evoked potentials (SSVEP) in the electroencephalogram (EEG) (Kabdebon et al., 2022, Norcia et al., 2015). The RVS method is particularly suited to measure infants’ visual overt and covert attention (Christodoulou et al., 2018), visual foraging (Robertson et al., 2012), and perception (Köster et al., 2017a, Köster et al., 2023a). This is due to its robustness to EEG artifacts, high signal-to-noise ratio (SNR) and the experimental control of the elicited brain response (Bánki et al., 2022, Kabdebon et al., 2022, Köster et al., 2023b, Peykarjou, 2022). Thus, we propose that the RVS method is well suited to assess social phenomena as well, such as joint attention in live social interaction studies. RVS can serve as a promising tool to counteract the issues of prior dual-EEG studies with adult-infant dyads. Precisely, it can track attention in a social interactional setting with high SNR while allowing to compute neural synchrony in the stimulated frequency band of interest.

Here, we applied this methodological approach to quantify the effects of communicative signals during joint attention on dyads’ brain activity and neural synchrony. In particular, RVS allowed us to track the co-occurrence of dynamic shifts in infants’ and caregivers’ visual attention when dyads observed flickering images of everyday animals and objects on a screen. The stimulation frequency was chosen correspondent to infants’ and adults’ theta oscillatory band, as theta oscillations in infants are implicated in anticipatory and sustained attention (Wass et al., 2018b, Xie et al., 2018), social attention (Jones et al., 2015, Orekhova et al., 2006), learning, encoding (Begus and Bonawitz, 2020, Köster et al., 2019, Köster et al., 2021), and cognitive ability (Braithwaite et al., 2020, Jones et al., 2020, Perapoch Amadó et al., 2023). In adults, theta oscillations are also involved in attention sampling (Fries, 2023, Klimesch, 2012, Köster and Gruber, 2022), learning (Lisman and Jensen, 2013), and memory (Klimesch, 1999). Across trials within the dyads, we manipulated communicative cues by instructing the caregiver to either communicatively engage with the infant by making eye contact, using IDS, and pointing to the images (joint attention condition) or quietly watch them together with the infant (joint watching condition).

Our first research question was whether communicative cues influence the neural responses of infants and caregivers during joint attention. We hypothesized that communicative signals during joint attention would enhance infants’ and mothers’ attention, reflected in increased individual neural responses (SSVEP amplitudes) in the joint attention compared to the joint watching condition. Previous research showed that SSVEPs of infants and adults are enhanced by attention: 3-month-olds’ SSVEP amplitudes increased due to attention modulation (i.e., watching rotating flickering objects compared to steady objects) (Robertson et al., 2012), and several studies with adults found an enhancing effect of attention on SSVEP amplitude (e.g., Gulbinaite et al., 2019; Morgan et al., 1996; Müller et al., 1998). Additionally, existing work with adults demonstrated that eye contact marks shared attention episodes (e.g., Wohltjen and Wheatley, 2021) and leads to changes in brain activity (e.g., Hirsch et al., 2017; Kompatsiari et al., 2021; Lachat et al., 2012; Noah et al., 2020; Szymanski et al., 2017). Our second research question was whether communicative signals impact infant-caregiver neural synchrony during joint attention. We expected that communicative cues, through establishing mutual reference (Hoehl et al., 2014, Hoehl and Bertenthal, 2021, Siposova and Carpenter, 2019), will promote the alignment of attention in the dyad leading to a higher degree of alignment between infants’ and caregivers’ neural responses (i.e., higher neural synchrony). This would be reflected in higher SSVEP amplitude envelope correlations (AECs) between the brain signals of infants and caregivers during joint attention compared to joint watching. Such an analysis could be informative on how communicative cues during joint attention shape mutual neural processes in the dyad beyond their effects on intra-brain attention dynamics only. Amplitude envelopes (AEs) capture energy fluctuations in neural oscillations over time, and AECs are obtained by correlating the amplitude envelopes of two brain signals to assess the mutual neural dynamics of two interacting partners (Ayrolles et al., 2021, Bruns et al., 2000, Marriott Haresign et al., 2022, Zamm et al., 2018, Zamm et al., 2023). Two brain signals display envelope coupling when their envelopes show corresponding patterns of change in amplitude over time (Zamm et al., 2023). When quantifying neural synchrony based on SSVEPs, it is more suitable to use correlation-based measures such as AEC instead of phase-based ones such as phase-locking. Since SSVEP is an evoked response phase-locked to an external visual stimulus, higher phase-synchrony could simply arise due to increased levels of phase resetting of the two brain signals to the common external stimulus, irrespective of interpersonal neural synchrony (Burgess, 2013, Marriott Haresign et al., 2022). In contrast, AECs can detect neural synchrony independent from phase coherence and are less susceptible to measurement jitter (Zamm et al., 2018, Zamm et al., 2021). Prior studies that investigated neural synchrony based on SSVEP data also used correlation-based measures (e.g., Painter et al., 2021).

In contrast to earlier EEG studies mainly focusing on infants’ brain responses during joint attention, this study provides new insights into how communicative cues dynamically modulate both infants’ and caregivers’ brain activity during joint attention.

## 2 Materials and methods

All study procedures and analyses reported here were pre-registered on AsPredicted^1^ in line with open science practices.

### 2.1 Participants

Participants were full-term born infants with typical development (*n* = 37; age in months: *M* = 12.11, *SD* = 0.61, range: 11.13–13.47; 18 girls) and their mothers (age in years: *M* = 34.3, *SD* = 4.86). In the sample, the number of infants was balanced between the two age groups of 11 months 0–30 days (*n =* 19) and 12 months 0–30 days (*n =* 14) or older. A small number of infants were 13 months 0–14 days old (*n* = 4). The study was approved by the Ethics Committee of the University of Vienna (Ref. 00455). Informed written consent before participation in the study was obtained from the mother. The study was conducted following the provisions of the World Medical Association Declaration of Helsinki.

Regarding attrition rates, 12 additional dyads were excluded from the analysis because infants did not comply with the EEG assessment (*n* = 3), did not provide a sufficient number of clean epochs (n = 7, see ‘EEG procedure’ for details), or because a technical error occurred during the EEG recording (*n* = 2). The initial sample size was calculated based on previous studies using a similar design (Köster et al., 2017a, Köster et al., 2023a).

### 2.2 Stimuli and procedure

Families were recruited from the database of the Children’s Studies Vienna at the University of Vienna for one experimental session that lasted approximately 60–90 min. In a brief warm-up task, mothers were shown printed images similar to the actual stimuli. First, an eye symbol was presented to the mothers who were asked to practice making short (1–3-s-long) eye contact with their infant while saying, ‘Let’s watch pictures together!’ Then an attention-getter (star) was presented, followed by an image (object/animal) printed in a frame. Mothers were asked to make a short comment in IDS (i.e., ‘Look at this!’; ‘Look at that!’) and point out the image with their index finger at the frame (without covering the object), to show it to their infant. Subsequently, two additional images were shown, and mothers were instructed to keep pointing to the frame around the object by leaving their index finger there. This warm-up phase served to train mothers to use communicative signals to establish joint attention with their infant later during the EEG task. To avoid fussiness during the EEG cap preparation, three alternating child-friendly cartoon videos (Sing mit mir - Kinderlieder, 2014, Sing mit mir - Kinderlieder, 2016, Sing mit mir - Kinderlieder, 2017) were played on a computer screen while infants either sat on a highchair to watch the cartoons or played with an experimenter and their mother with age-appropriate toys on a play carpet.

**Fig. A1:**
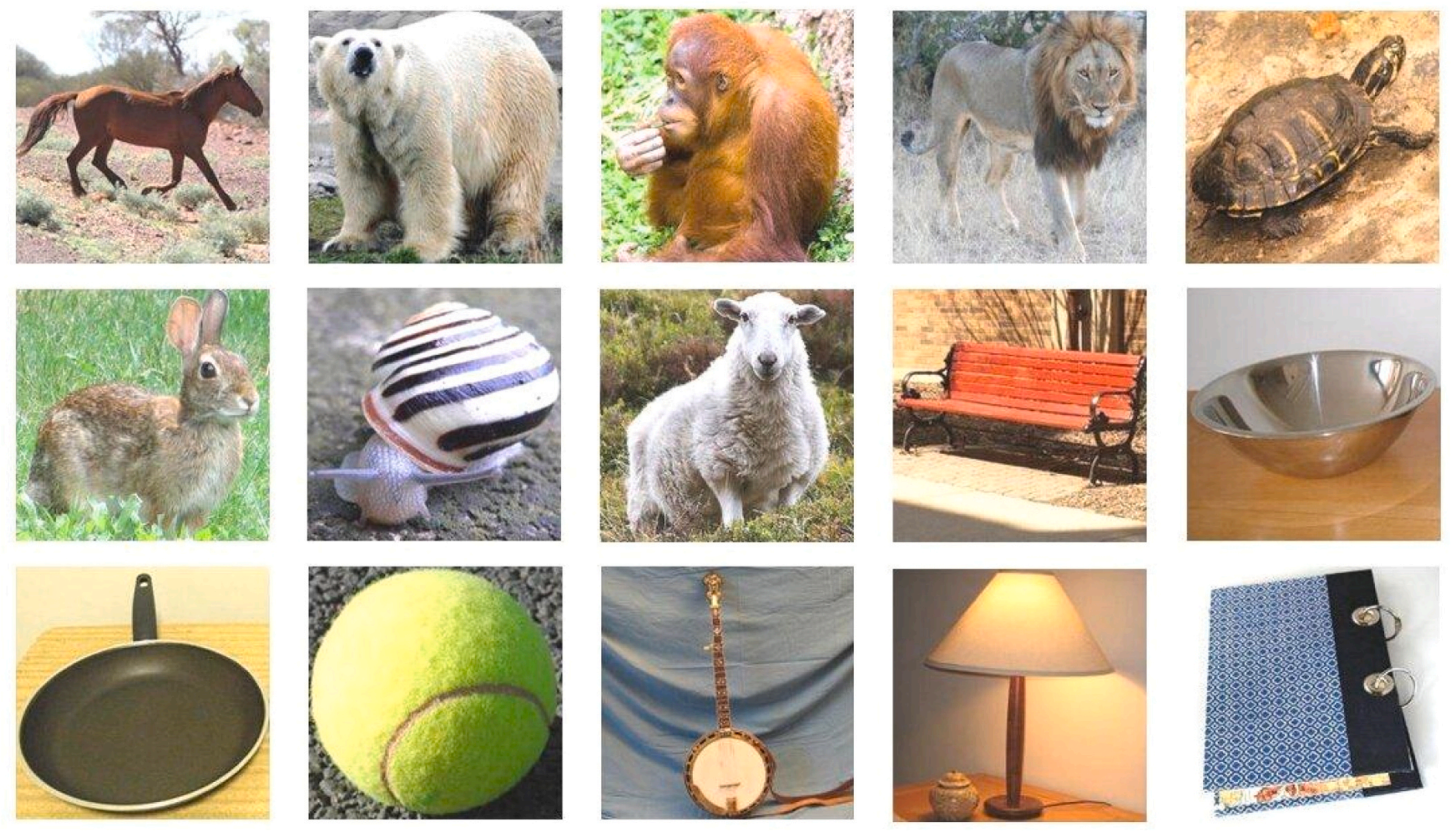
**Overview of all stimuli images.** Fifteen natural images of animals and objects in front of a natural background were presented (retrieved from Cichy et al., 2016) in each condition (JA and JW) four times (twice per block, without consecutive repetitions), each image flickering at 4 Hz for 2 s. Image order was randomized within-dyad (within conditions between blocks, between conditions) and between dyads.

In the EEG paradigm, dyads saw 15 natural images of everyday animals (*n* = 8) and objects (*n* = 7) in their natural environment (e.g., a horse in the field, or a bench on the street, retrieved from Cichy et al., 2016, see Appendix A, Fig. A1), while their brain activity was simultaneously recorded with mobile EEG (Smarting, mBrainTrain, Serbia). Images were matched in luminance between categories (animals, objects), *t*(13) = 0.30, *p* = .77, *d* = .16.

The EEG session included two conditions: joint attention (JA) with communicative signals and joint attention without communicative engagement, in the following referred to as joint watching (JW). The conditions were alternately presented in four blocks, and the condition order was counterbalanced across dyads (i.e., JA-JW-JA-JW or JW-JA-JW-JA). Of the final sample (*n* = 37), 20 dyads (54%) started with the JA, whereas 17 dyads (46%) started with the JW condition. Before the session, mothers were asked to complete a sociodemographic questionnaire via the online tool SoSci Survey (Leiner, 2019) at www.soscisurvey.de. Alternatively, a printed-out version of the survey was filled out before or after the session.

The Psychophysics Toolbox (Brainard, 1997, Kleiner et al., 2007, Pelli, 1997, Version 3.0.) in MATLAB (MathWorks Inc., US, Version R2018b) was used for stimulus presentation on a cathode-ray tube (CRT) computer screen (Vision Master Pro 454, Iiyama Corporation, Japan).

### 2.3 EEG procedure

Dyads sat in front of a computer screen at a distance of 60 cm. Mothers sat on a regular chair while infants were seated in a Stokke Tripp Trapp highchair with seat and back pillows. Infants occasionally were moved to sit on the mother’s lap during the task to avoid fussiness (see section 3.1. ‘Control analyses’). We applied a within-dyad design with two conditions (JA, JW) presented alternately in four blocks. In each block, dyads observed the same 15 images depicting a natural object (animal/everyday object) in front of a natural background (for all pictures, see Appendix A, Fig. A1). Each image was shown twice per block (30 images), thus four times per condition (60 images). This design resulted in 120 stimulus images presented to each dyad. Within a block, images were presented without consecutive repetitions, and image order was randomized across blocks, conditions, and dyads. Images were shown for 2 s each, preceded by a 1-s attention-getter (a yellow star accompanied by a short sound) and a black screen with a variable duration of 0.5–0.8 s for recording EEG baseline activity and providing a brief pause between flickering stimuli. For the attention-getter, five different sounds with varying duration (0.5–1 s) were used in a randomized order across images, conditions, and dyads to direct infants’ attention to the stimuli on the screen throughout the task.

**Fig. 1:**
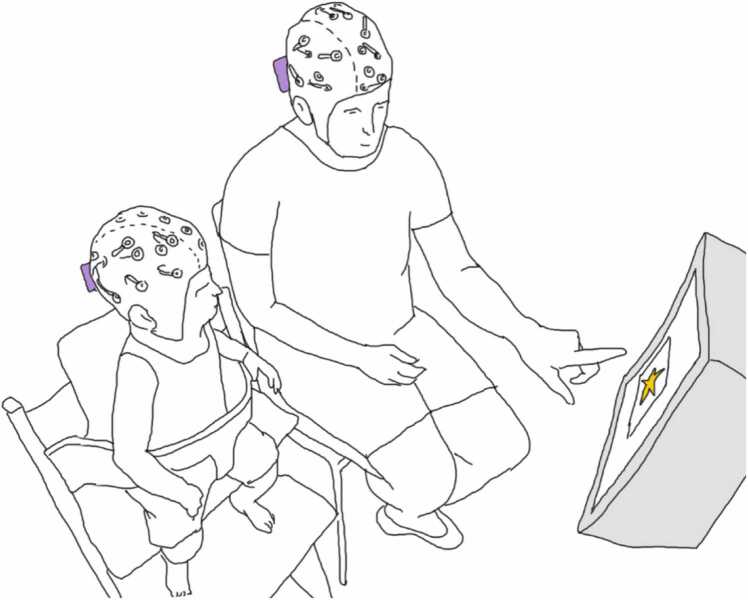
**Recording set-up.** Mother-infant dyads watched images preceded by an attention getter (shown in yellow) on a computer screen (shown in grey) while their brain activity was recorded simultaneously with mobile EEG (amplifiers depicted in purple). In the JA condition (shown here), the mother made eye contact with the infant, said a verbal comment in IDS, and pointed to the images appearing on the screen. In the JW condition, the dyad watched the images without interaction.

In both conditions, every third image was preceded by a so-called ‘pre-phase’ for 3 s. In the JA condition's pre-phase, an eye symbol (11.7 × 6.5 cm) was shown for 3 s. During this time, mothers had to engage in eye contact with their infant sitting next to them and say the phrase: ‘Let’s watch pictures together’ in IDS. When the attention-getter with the sound appeared on the screen, mothers were instructed to point at the screen with their index finger, particularly to a rectangle frame (16.3 × 15 cm) that surrounded the attention-getter star (9.3 × 10.5 cm) and was slightly larger than the upcoming stimuli images (14.4 × 13.8 cm). While pointing, mothers were also asked to make another short comment in IDS (i.e., ‘Look at this!’ in German [’Schau da!’], written as a reminder over the attention-getter) and then keep pointing by leaving their finger on the screen (see Fig. 1 for the recording set-up). The first image in the trial was shown, after which two subsequent images appeared, each preceded by the attention getter (with the text ‘Say nothing’ in German [‘Nichts sagen’]) and the black screen baseline (0.5–0.8 s). Mothers were instructed to leave their index finger on the screen for the entire trial duration (3 images) without retracting it from the screen.

**Fig. 2:**
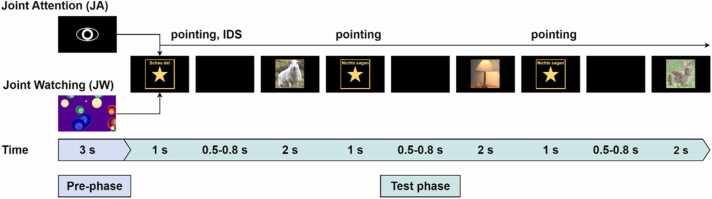
**Trial structure.** Each trial consisted of a 3-s pre-phase and a 10.5–11.4-s test phase. In the JA pre-phase, an eye symbol was displayed on the screen for mothers to engage in eye contact with the infant. In the JW pre-phase, an attention-grabbing video of colorful bubbles was shown. In the test-phase, stimuli were identical between conditions and included three images of animals/objects in front of a natural background, flickered at 4 Hz for 2 s each. Each image was preceded by a 1-s attention-getter star with condition-specific instructions for the mother (i.e., in the JA condition, ‘Schau da!’ to prompt a verbal comment in IDS for the first image, and ‘Nichts sagen’ for the following two images as shown here, and in JW, 'Nichts sagen' for all three images). Next, a short black screen with a random duration (0.5–0.8 s) was shown before each image for EEG baseline activity recording. In JA trials, mothers were asked to point at the screen from the first attention-getter, make the verbal comment in IDS, and leave their finger on the screen until the last image's offset. In JW trials, mothers watched the stimuli on the screen without communicative engagement with the infant.

In the JW condition's pre-phase, a non-social attention-enhancing video (animated video of colorful, moving bubbles accompanied by a popping sound) was shown for 3 s. Next, three images were shown, each preceded by the attention-getter (with the text ‘Say nothing’ in German [‘Nichts sagen’]) and the black screen for baseline recording, constituting one trial. For the JW condition, mothers were instructed to watch the pictures quietly the whole time without any communicative engagement with their infant. The trial structure is illustrated in Fig. 2.

Between blocks, mothers saw written instructions on the screen as a reminder about their task in the upcoming block. In the JA condition, the following text was displayed: ‘Break. In the next part, look at your child when you see the eye, then point at the screen!’ (In German: ‘Pause. Im nächsten Teil schauen Sie, wenn sie das Auge sehen, zu Ihrem Kind, danach zeigen Sie auf den Bildschirm!’) In the JW condition, the following text was displayed: ‘Break. In the next part, just look at the screen!’ (In German: ‘Pause. Im nächsten Teil schauen Sie nur auf den Bildschirm!’).

We applied rhythmic visual stimulation (Kabdebon et al., 2022, Köster et al., 2023b, Martens et al., 2011, Müller et al., 2003) to track infants’ and mothers’ attention (i.e., neural responses) to the images. To elicit SSVEPs for the images, all stimuli images were flickered at 4 Hz. This was achieved by controlling the presentation of an 80 Hz CRT monitor (Vision Master Pro 454, Iiyama Corporation, Japan) at every refresh cycle, as established in our previous work (Köster et al., 2017a, Köster et al., 2023a). For a flicker rate of 4 Hz, the images were presented at a duty cycle of 2:2, i.e., two refresh cycles with the images being illuminated (100% brightness) and two refresh cycles with the images being darkened (10% of the original brightness). Images were presented at a visual angle of 9–13.5 × 9–13.5° on a 19-inch (18-inch viewable) monitor with 1024 × 768 resolution and a refresh rate set to 80 Hz. In case infants’ attention decreased during the stimulus presentation, a child-friendly animation (a black spiral [8 × 8 cm] turning in front of a white background [22.5 × 17.3 cm] accompanied by music) was shown in between images by the experimenter. In case of infant fussiness, the presentation was paused or stopped. Dyads were video recorded during the EEG assessment for the subsequent coding of infants’ and mothers’ gaze behaviour, mutual eye contact, and maternal pointing.

### 2.4 EEG apparatus

The neural activity of infants and mothers was recorded simultaneously with EEG hyperscanning (Nguyen et al., 2020, Norton et al., 2022, Turk et al., 2022, Wass et al., 2020b), applying two Smarting mobile EEG systems (mBrainTrain, Serbia) with 24 Ag/AgCl scalp electrodes (EasyCap GmbH., Germany). Electrodes were positioned according to the international 10–20 layout system, using the ‘standard natural layout’, with the reference electrode positioned at the FCz site and the ground electrode at the FPz site. During EEG cap preparation, the scalp under each electrode was cleaned twice with 70% isopropyl alcohol using cotton swabs. A customized electrolyte gel was applied twice for each electrode using curved-tip syringes to ensure that impedances remained under 10 kΩ. To sustain high signal quality, the Bluetooth dongles for the wireless EEG systems were positioned right next to the participants using two USB 2.0 extension cables. Mobile EEG amplifiers were connected to the PC via the BlueSoleil software (IVT Corporation, Version 10.0.498.0). EEG was recorded at a sampling rate of 500 Hz using amplifiers with 3D built-in gyroscopes and the Smarting Streamer application (mBrainTrain, Serbia, Version 3.4.3). EEG markers were generated automatically at the start of each pre-phase (in JA: at the eye symbol image onset; in JW: at the bubble video onset), at each stimulus image onset, and at the end of the procedure using the Lab Streaming Layer (LSL, Version 1.13.0-b3) data acquisition and synchronisation framework in MATLAB (MathWorks Inc., US, Version R2018b). Markers were sent to the Smarting Streamer application on the presentation PC. The two EEG streams were streamed on two separate computers (presentation and recording PCs) with the help of the two Bluetooth dongles (one connected to each PC). The two PCs were connected with a LAN cable and the Smarting Streamer application was configured to pass the firewall of the recording PC. This allowed us to record both EEG streams and the marker stream into one EEG file on the recording PC.

### 2.5 EEG pre-processing and analyses

All EEG data analyses, and visualization were conducted in MATLAB (MathWorks Inc., US, Version R2018b) using the EEGLAB toolbox (Delorme and Makeig, 2004, Version 2019) and custom-made scripts. Statistical analyses were conducted in JASP (JASP Team, 2023, Version 0.17.1) and RStudio (Posit team, 2023) using the ‘lme4’ (Bates et al., 2015), ‘lmerTest’ (Kuznetsova et al., 2017), and ‘emmeans’ (Lenth, 2020) packages. For statistical data visualization, RStudio and the Raincloud-Shiny online plotting application (Allen et al., 2019) were used.

#### 2.5.1 EEG pre-processing

Before EEG analysis, continuous EEG data were pre-processed using the EEGLAB toolbox (Delorme and Makeig, 2004; Version 2019). First, data were band-pass filtered from 1 Hz to 48 Hz and segmented into epochs based on stimulus onset and image presentation length (1000 ms before and 3000 ms after image onset) to provide a time window suitable for subsequent wavelet analysis (Cohen, 2014). The mean baseline activity in the 500 ms time window before image onset was subtracted from all channels. Next, we visually inspected the epoched data, removed noisy epochs, and interpolated noisy channels with spherical interpolation. The presence of noise in the epochs was also determined by visually inspecting data from the three gyroscope channels, which were then excluded before independent component analysis (ICA). Eye blinks and muscle artifacts were detected using an ICA procedure and removed after visual inspection (Chaumon et al., 2015). Following the ICA procedure, the EEG data was again visually inspected and any remaining noisy epochs were removed, whereas any remaining noisy channels were interpolated (up to a maximum of four scalp channels per participant over the course of the whole pre-processing). Further, epochs corresponding to images when mothers and infants did not look at the screen from image onset (or at least 500 ms within the onset, see ‘Video coding’) until the end of image presentation (2000 ms) were removed based on video coding data before ICA (if such epochs contained visible noise) or afterwards (if they were not visibly noisy). At this point, infants and mothers with less than five clean epochs per condition were excluded from further analyses. The number of clean epochs for the included infants and mothers were compared between conditions using dependent *t*-tests (see section 3.1. ‘Control analyses’). In the following, EEG signals were re-referenced to the average of all scalp electrodes, the original reference (FCz) was removed, and then epochs were split into conditions (JA and JW).

#### 2.5.2 Time-frequency analysis of SSVEPs

Evoked spectral power over time was obtained by averaging EEG data across epochs. The resulting event-related potentials were analysed using Morlet wavelets (Tallon-Baudry and Bertrand, 1999) with seven cycles, in 0.5 steps, in a frequency range of 1–15 Hz (resulting in 29 frequency bands) and a time window of − 1000 and 2500 ms, to obtain SSVEP power values in the frequency of interest (4 Hz).

In the first step, to verify if the stimulation frequency (4 Hz) elicited SSVEP responses, we calculated the time-resolved SNR of SSVEP power for infants and mothers across epochs from each condition separately. For each participant, for each frequency band, channel and time point (i.e., the result from the wavelet analysis), the spectral power at a given time point was divided by the average power of the surrounding frequencies (−2, −1, +1, +2 Hz, around the target frequency) averaged across the wavelet analysis time window (−1000 to 2500 ms), as a proxy for the noise level, and one was subtracted. The subtraction of one from the ratio was performed to adjust for the fact that the denominator of the SNR ratio is a measure of the noise level in the signal, and the obtained SNR is, therefore, relative to this noise level. The resulting SNRs were averaged across all channels (C3, C4, CPz, Cz, AFz, F3, F4, F7, F8, Fp1, Fp2, Fz, M1, M2, O1, O2, POz, P3, P4, P7, P8, Pz, T7, T8) and all participants to obtain the grand mean SNR per condition. To statistically test whether we successfully elicited SSVEP responses in infants and mothers, we used dependent *t*-tests, comparing these condition-level grand mean SNR values against the noise level of 0 at 4 Hz. This analysis revealed a peak at 4 Hz for the driving frequency for infants and mothers in each condition, JA and JW (see section 3.1. ‘Control Analyses’). The wavelet analysis approach applied here corresponds to the one used in our previous studies (Köster et al., 2017a; Köster et al., 2023a). SSVEP SNR values were calculated with the neighboring frequencies instead of the pre-stimulus baseline activity due to fewer epochs and a lower stimulation frequency, similar to prior RVS studies with infants (Christodoulou et al., 2018, Robertson et al., 2012) and adults (Sciortino and Kayser, 2023).

#### 2.5.3 Intra-brain analyses (individual SSVEPs)

For the topographies, for both infants and mothers, we calculated the condition-specific grand mean signal for 4 Hz (individual SNR values) averaged over image presentation duration (0–2000 ms) in each condition. We also computed topographic maps for the grand mean signal per each condition and for the condition difference in the grand mean signal during this time window. For all subsequent analysis steps, we first calculated the individual SSVEP SNR values at 4 Hz for each time point during the image presentation (0–2000 ms) and for each channel (except for mastoid channels M1 and M2). To explore the data, the SSVEP SNR values were first averaged across all individual electrode sites (except at mastoid channels M1 and M2) and compared between conditions within-subject (for infants and mothers separately) with dependent *t*-tests. The same tests were performed for scalp-region-specific electrode sites identified based on visual inspection. Next, to assess condition differences at scalp-region-specific electrode sites, we pre-defined the following regions of interest, namely central: C3, C4, CPz, Cz; frontal: AFz, F3, F4, F7, F8, Fp1, Fp2, Fz; occipital: O1, O2, POz; parietal: P3, P4, P7, P8, Pz; and temporal: T7, T8. These regions were pre-selected based on findings from previous SSVEP studies with infants (Köster et al., 2019, Köster et al., 2023a). For each channel in these regions, condition-specific SSVEP SNRs at 4 Hz were averaged across the whole time window of image presentation length (0–2000 ms) per participant. Then we conducted a linear mixed effects model (LMM) with condition and region (central, frontal, occipital, parietal, temporal) as fixed effects, individual as random effect, and the average SSVEP SNR per channel as the dependent variable. To obtain *p* values we used the Satterthwaite estimate of denominator degrees of freedom (ddf). To account for multiple comparisons, pairwise contrasts were computed and *p* values were adjusted using multiple *t*-distribution. Finally, to test the spatial, temporal and spatial-temporal aspects of potential condition differences in SSVEP SNR values within-subject, we additionally used cluster-based permutation tests in the channel, time and channel-time dimensions (Maris and Oostenveld, 2007) implemented in the FieldTrip toolbox (Oostenveld et al., 2011) in MATLAB (MathWorks Inc., US, Version R2018b). For the channel dimension, we conducted permutation tests on time-averaged SSVEP SNRs across the whole time window (0–2000 ms) with cluster inclusion criterion of *p* < .05, 1000 Monte Carlo iterations, with dependent samples *t*-tests and with a critical alpha value of 0.25 for two-sided tests (as per Köster et al., 2017b). Cluster statistic was calculated as the sum of the *t* values of neighboring electrodes (with a minimum number of one channel). The significance of the cluster statistic was computed from a combined permutation distribution obtained from 1000 Monte Carlo iterations with randomly assigned JA and JW conditions. This resulted in cluster-wise *p* values unaffected by inflated false-positive rates otherwise arising from multiple comparisons.

For the time dimension, we performed separate permutation tests on channel-averaged SSVEP SNRs from each pre-defined scalp region (i.e., central, frontal, occipital, parietal, temporal plus central-occipital-parietal channels for infants, and central-frontal-parietal channels for mothers, see 'SSVEP topographies') over the whole time window (0–2000 ms). For this, we used the ‘permutest’ function (Version 1.0.0, Gerber, 2023) with two-sided dependent samples tests with 1000 permutations and a *p* value threshold of 0.025 based on Oostenveld et al. (2011). For the channel-time dimension, we used the SSVEP SNR data over the whole time window (0–2000 ms, unaveraged), and applied the function ‘ft_timelockstatistics’ (Oostenveld et al., 2011), and the same testing parameters and criteria as for the channel dimension.

**Fig. A2:**
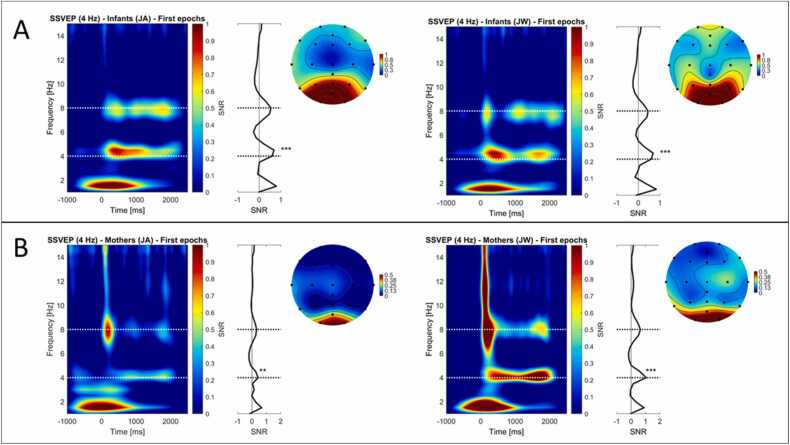
**SSVEP SNR control analysis results – Time-frequency plots of grand mean SSVEP SNRs in the first epochs.** SNR values averaged across all infants (A) and mothers (B) who provided at least one first epoch per condition Averaging was across all first epochs (per condition) and all electrodes (C3, C4, CPz, Cz, AFz, F3, F4, F7, F8, Fp1, Fp2, Fz, M1, M2, O1, O2, POz, P3, P4, P7, P8, Pz, T7, T8), displayed on the adjacent EEG topographic maps (except M1, M2). Grand mean SNR values were significantly different from noise level 0 at the stimulation frequency of 4 Hz for infants and mothers in each condition (JA shown on the left, JW on the right) for the first epochs, with all *p* values < .001 (denoted with ***) except for mothers in JA: *p* < .01 (denoted with **). Note that scale limits are adjusted to the average SNR levels in this smaller subset of first epochs (thus are lower than in Fig. 3).

As an exploratory analysis step, we performed the same analyses only for the subset of the first epochs from both conditions (see Appendix B, Fig. A2). This was to control for the fact that in the JA condition, the first epochs might have been more engaging for infants, as they included all three communicative cues (eye contact, pointing, IDS) whereas the subsequent two epochs were only accompanied by pointing. We conducted dependent *t*-tests on the SSVEP SNR data averaged across electrodes and image presentation time separately for infants and mothers. We also conducted identical analyses as for the complete dataset (of all epochs): we ran the same LMMs (see Appendix B, Tables A1–6.) and performed the same cluster-based permutation tests in the time dimension and channel-time space to account for any condition differences specifically in these first epochs (see Appendix B).

#### 2.5.4 Inter-brain analyses (AECs)

For the AEC analysis, we included 31 out of the 37 dyads^2^ into the analysis who had at least five clean EEG epochs that both infant and mother attended (based on video coding and EEG pre-processing) in each condition, referred to as ‘mutual epochs’. For the included infants and mothers, the number of mutual epochs was compared between conditions using dependent *t*-tests (see section 3.1. ‘Control analyses’). To verify if SSVEP responses were significantly different from the noise level during mutual epochs, we first extracted evoked spectral power over time by averaging EEG data across these epochs and analysed them with Morlet wavelets, as described above. We extracted amplitude values (instead of power) from this analysis and calculated the time-resolved SNR of SSVEP amplitude for infants and mothers for each condition separately. We performed the same control analyses and visualization of the grand mean SSVEP SNR described above for the data from all epochs. This analysis revealed a peak at 4 Hz for the driving frequency for infants and mothers in each condition, JA and JW (see section 3.1. ‘Control Analyses’). Next, we analysed data from each mutual epoch separately with Morlet wavelets (without averaging across epochs) to obtain SSVEP amplitude values for each mutual epoch, time point, and channel per participant and condition. This approach was similar to prior studies that obtained AEs with Morlet wavelet analysis to compute AEC as a measure of intra-brain connectivity (e.g., Hipp et al., 2012; Shah-Basak et al., 2022). Within-subject and condition, resulting AEs were first baseline corrected (subtracting a pre-stimulus baseline, in the time window of 500–200 ms before image onset) and averaged across all channels (except mastoids M1, M2) per epoch.

For each dyad, Pearson correlation was used to correlate the AEs of infants and mothers within each mutual epoch. The obtained correlation values were converted to Fisher’s *z* values to ensure normality and averaged across epochs within conditions (Zamm et al., 2018, Zamm et al., 2021, Zamm et al., 2023). This resulted in a single correlation value representing the mean neural synchrony of EEG AEs for each dyad per condition. These AEC values were compared within-dyad with a dependent *t*-test. The same analysis of AECs was performed for scalp-region-specific channels. To account for variability between dyads and within dyads between epochs, we compared epoch-level AECs (i.e., *z* values) between JA vs JW averaged across all channels, and occipital channels. For this we used LMMs with condition as a fixed effect and dyad as a random effect.

Additionally, we calculated channel-by-channel AECs for each epoch, and Fisher *z*-transformed the values before averaging across epochs within dyad. Then we conducted a permutation test with cluster inclusion criterion of *p* < .05, 1000 iterations, with dependent samples *t*-test. The test computed the *t* value for each of the electrode pairs, comparing the two conditions within dyad. The same procedure was repeated in 1000 random permutations of the original data, shuffling condition labels within dyad. For each permutation, the largest *t* value was obtained to form a nonparametric estimate of the distribution of the largest *t* value under the null hypothesis that conditions are not different. *P* values were computed for each electrode pair in the original *t*-map as the proportion of permutations that resulted in a comparison with a larger *t* value than the comparison in question. Finally, we applied a false discovery rate (FDR) correction to the obtained *p* values to adjust for multiple comparisons.

### 2.6 Video data coding and analysis

Dyads were video recorded during the EEG task with an action camera (SONY FDR-X3000 4K Action Cam with Live View Remote, Sony Corporation, Japan) at 60 frames per second for subsequent video coding of infants’ and mothers’ gaze behaviour, mutual eye contact, and maternal pointing. Video annotation was performed using Interact (Mangold International GmbH., Germany, 2018) to mark epochs corresponding to images when infants and mothers did not look at the screen for the entire image presentation duration of 2000 ms. These epochs were later excluded from the EEG analysis, except if infants and mothers looked at the screen within 500 ms (30 frames) from image onset and looked continuously until the image's offset. Mutual eye contact was coded for the pre-phase of the JA trials when the mother and infant looked at one another. Additionally, maternal pointing was coded during the image presentations as missed pointing in the JA condition or accidental pointing in the JW condition to control for mothers’ compliance with the instructions. To assess the precision of maternal instruction following (i.e., pointing within condition), we descriptively assessed the frequencies of missed pointing in JA, and accidental pointing in JW based on the video coding. In JA, mothers missed pointing only at 8% of the images on average (*SD* = 16%, range: 0–65%). In JW, mothers accidentally pointed only at 2% of the images on average (*SD* = 4%, range: 0–15%).

We established high inter-rater reliability between two independent coders who coded 27% of the video data (10 dyads drawn from the included sample of *n* = 37) for infants' gaze (κ = 0.82), mothers’ gaze (κ = 0.72), and frequency of mutual eye contact during JA (κ = 0.88). Inter-rater reliability for mothers’ missed pointing during JA and accidental pointing during JW was moderate (κ = 0.62). The number of attended images (referred to as ‘gaze behaviour’) was compared between conditions (JA vs JW) both for infants and mothers using dependent *t*-tests (see section 3.1. ‘Control analyses’). All video data analyses were conducted in JASP (JASP Team, 2023, Version 0.17.1). Inter-rater reliability analysis was performed in RStudio (Posit team, 2023) using the ‘irr’ package (Gamer et al., 2019).

## 3 Results

### 3.1 Control analyses

#### 3.1.1 Behavioral interactional data

To assess condition differences in gaze behaviour, we compared the number of attended images between conditions separately for infants and mothers. Based on the video coding of gaze data, infants attended on average 65% (*SD* = 11.6%, range: 38–85%) of the total number of presented images in the JA and 54% (*SD* = 13.5%, range: 24–87%) in the JW condition. This difference in gaze behaviour (i.e., more attended trials in JA vs JW) was significant between conditions, *t*(36) = 5.37, *p* < .001. Mothers attended, on average, 78% (*SD* = 20%, range: 23–100%) of the total number of presented images in the JA and 79% (*SD* = 23%, range: 10–100%) in the JW condition. There was no significant difference in mothers’ gaze behaviour between conditions, *t*(36) = −0.4, *p* = .69. To account for condition differences in mutual eye contact (i.e., manipulation check), we compared the frequency of mutual eye contact between conditions, coded from the videos. Mutual eye contact between infant and mother was established in 30% of the JA pre-phases on average (*SD* = 29%, range: 0–95%) and in 0% of the JW pre-phases. To assess condition differences in infants’ seating position, the frequency of infants sitting on the mother’s lap was compared between conditions. Among all infants, 30% of them (*n* = 11) always sat in a highchair throughout the task, while 70% of the infants (*n* = 26) preferred to sit on the mother’s lap for some time of the task. Overall, infants in the full sample sat on the mother’s lap on average 37% of the time during images (*SD* = 33.51%, range: 0–100%). For the subgroup of infants, who spent at least some time sitting on the mother’s lap, this was on average 53% of the time during image presentation (*SD* = 27%, range: 11–100%). In JA, this subgroup of infants sat on the mother’s lap on average 51% of the time (*SD* = 34%, range: 10–100%) whereas in JW, 55% of the time (*SD* = 29%, range: 10–100%). There was no significant difference in the frequency of infants sitting on the mother’s lap between conditions in the full sample, *t*(36) = −0.6, *p* = .53.

#### 3.1.2 EEG data quality

To assess condition differences in EEG data quality for both the intra- and inter-brain analyses, we compared the number of artifact-free epochs between conditions separately for infants and mothers. Infants included in the SSVEP SNR (intra-brain) analysis provided on average 33% (*M* = 19.6, *SD* = 7.3, range: 9–37) artifact-free epochs in the JA, and 27% (*M* = 16.2, *SD* = 5.7, range: 6–29) epochs in the JW condition, while mothers provided 48% (*M* = 28.1, *SD* = 10.3, range: 6–50) artifact-free epochs in the JA, and 67% (*M* = 38.4, *SD* = 12.6, range: 5–57) epochs in the JW condition. Infants had a significantly higher number of such epochs in the JA vs the JW condition, *t*(36) = 2.72, *p* = .01, whereas mothers had significantly more epochs in the JW vs the JA condition, *t*(36) = −5.89, *p* < .001. Dyads included in the AEC (inter-brain) analysis provided on average 19% (*M* = 12.1, *SD* = 5.5, range: 5–24) mutually attended, artifact-free epochs in the JA, and 21% (*M* = 13.7, *SD* = 6.1, range: 5–28) epochs in the JW condition. The number of mutually attended epochs was not significantly different between conditions, *t*(30) = −1.32, *p* = .20.

#### 3.1.3 SSVEP SNRs

**Fig. 3:**
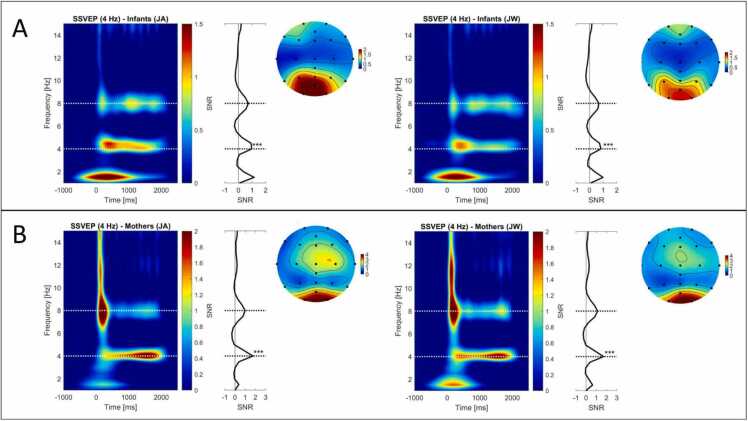
**Time-frequency plots of grand mean SSVEP SNRs.** SNR values averaged across all infants (A) and all mothers (B), across all epochs per condition and all electrodes displayed on the adjacent EEG topographic maps (except M1, M2). Grand mean SNR values were significantly different from noise level 0 at the stimulation frequency of 4 Hz for infants and mothers in each condition (JA shown on the left, JW on the right), with all *p*s < .001 (denoted with ***).

To statistically test whether we successfully elicited SSVEP responses in infants and mothers, we compared the condition-level grand mean SNR values (averaged across all participants, all epochs, and all electrodes) against the noise level of 0, at 4 Hz. Grand mean SNR values averaged across all electrodes (C3, C4, CPz, Cz, AFz, F3, F4, F7, F8, Fp1, Fp2, Fz, M1, M2, O1, O2, POz, P3, P4, P7, P8, Pz, T7, T8, see Fig. 3), and the whole image presentation duration (0–2000 ms) were significantly different from noise level during JA for infants, *t*(36) = 9.3, *p* < .001, and mothers, *t*(36) = 7.22, *p* < .001, as well as during JW for infants, *t*(36) = 7.56, *p* < .001, and mothers, *t*(36) = 6.94, *p* < .001 (Fig. 3).

**Fig. A3:**
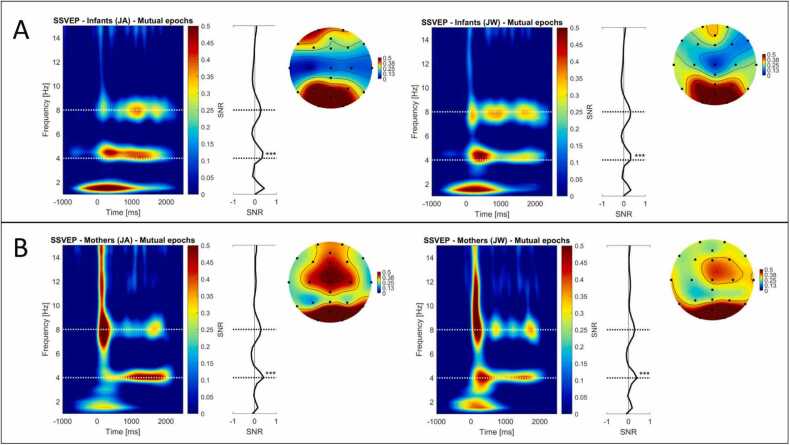
**AEC control analysis results – Time-frequency plots of grand mean SSVEP SNRs in mutual epochs.** SNR values averaged across all infants (A), and all mothers (B), across all mutual epochs included in the AEC analysis (per condition) and all electrodes (C3, C4, CPz, Cz, AFz, F3, F4, F7, F8, Fp1, Fp2, Fz, M1, M2, O1, O2, POz, P3, P4, P7, P8, Pz, T7, T8) displayed on the adjacent EEG topographic maps (except M1, M2). Grand mean amplitude SNR values were significantly different from noise level 0 at the stimulation frequency of 4 Hz for infants and mothers in each condition (JA shown on the left, JW on the right) for mutual epochs included in the AEC analysis, with all *p*s < .001 (denoted with ***). Note that scale limits are adjusted to the average SNR levels in this smaller subset of mutually attended epochs (thus are lower than in Fig. 3), and SNR was calculated for SSVEP amplitude vs power.

Before the AEC analysis, we statistically tested if SSVEP amplitude SNRs differed from noise level during the subset of epochs mutually attended by infants and mothers (see Appendix C, Fig. A3). This was to ensure that SSVEP SNR data quality was sufficiently high in this smaller subset of mutually attended epochs for both infants and mothers, before calculating AEs and AECs. We compared the condition-level grand mean amplitude SNR values (averaged across participants included in the AEC analysis, all mutually attended epochs per dyad, and all electrodes) against the noise level of 0 at 4 Hz. Grand mean SNR values averaged across all electrodes and the whole image presentation duration (0–2000 ms) were significantly different from noise level during JA for infants, *t*(30) = 7.76, *p* < .001, and mothers, *t*(30) = 5, *p* < .001, as well as during JW for infants, *t*(30) = 6.12, *p* < .001, and mothers, *t*(30) = 7.27, *p* < .001 (Fig. A3).

### 3.2 The effect of communicative signals on individual neural responses during joint attention: SSVEP findings

#### 3.2.1 SSVEP topographies

**Fig. 4:**
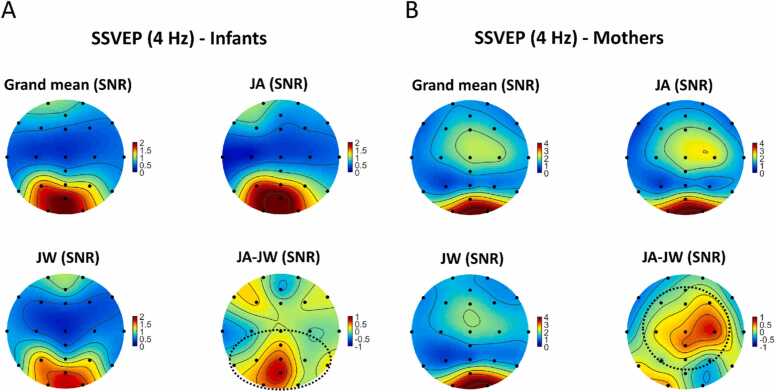
**Topographic maps of grand mean, condition-level, and condition difference SNRs (4 Hz).** Topographic maps for infants (A) and mothers (B) showing SSVEP SNR values at 4 Hz averaged across participants: between conditions (grand mean), for each condition (JA, JW), and the condition difference (JA-JW; the difference between the JA and JW SNRs). For the JA-JW maps, electrodes with an increased SNR during JA vs JW are marked with black dashed lines (central-occipital-parietal channels CPz, Cz, O1, O2, POz, P3, P4, P7, P8, Pz for infants, and central-frontal-parietal channels C3, C4, CPz, Cz, AFz, F3, F4, Fz, P3, P4, Pz for mothers).

**Fig. 5:**
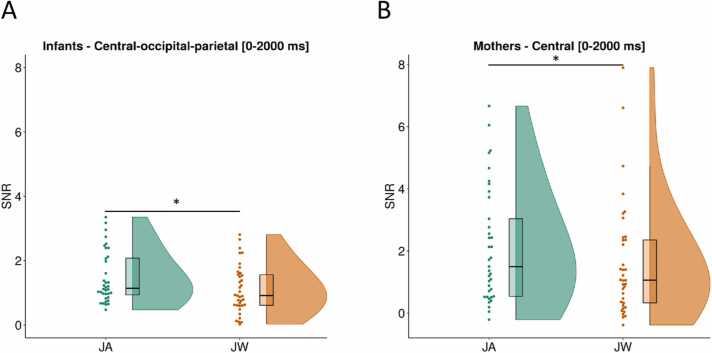
**SSVEP SNR (4 Hz) condition differences.** SSVEP SNRs at 4 Hz compared between conditions JA (in green) and JW (in orange) for infants (A) at central-occipital-parietal channels (CPz, Cz, O1, O2, POz, P3, P4, P7, P8, Pz), and for mothers (B) at central channels (C3, C4, CPz, Cz) during the whole duration of image presentation (0–2000 ms). SSVEP SNRs averaged across these channels were significantly higher during JA vs JW for infants, *p* = .03, and for mothers, *p* = .04 (marked with *).

**Fig. 6:**
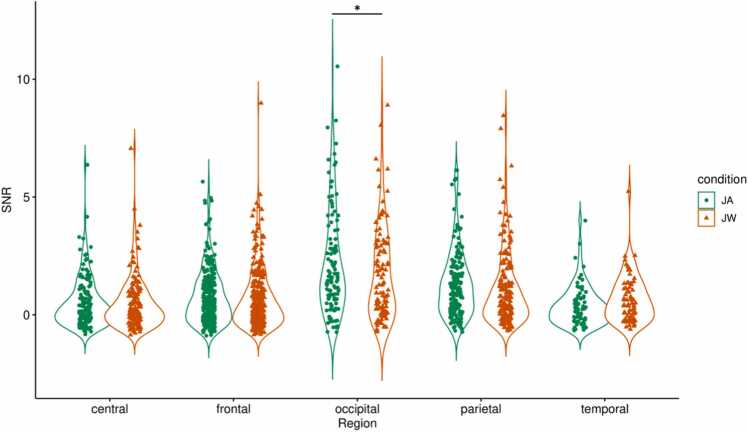
**LMM results - Infant SSVEP SNR (4 Hz) condition differences per scalp region.** SSVEP SNRs at 4 Hz were compared between conditions JA (in green) and JW (in orange) for infants at central, frontal, occipital, parietal, and temporal regions during the whole duration of image presentation (0–2000 ms). SSVEP SNRs in the occipital region were significantly higher during JA vs JW, *p* = .02 (denoted with *).

For infants, SSVEP SNRs for the whole image presentation duration (0–2000 ms) averaged across all channels were not significantly different between conditions, *t*(36) = 0.98, *p* = .33 (uncorrected). As an exploratory analysis, we visually inspected the condition-specific EEG topographic maps and their difference (Fig. 4**A**), and selected a set of central, occipital, and parietal channels (CPz, Cz, O1, O2, POz, P3, P4, P7, P8, Pz) for comparison (marked with a dashed line on Fig. 4**A**, in the following referred to as ‘central-occipital-parietal channels’). SNRs averaged across these channels were significantly higher during JA vs JW, *t*(36) = 2.25, *p* = .03 (uncorrected, Fig. 5**A**). In the LMM, when SSVEP SNRs during the whole image presentation (0–2000 ms) were compared between conditions and scalp-region specific channels of interest, there was no significant overall effect of condition, *F*(1,1582) = 2.29, *p* = .13, but a significant effect of region, *F*(4,1582) = 56.42, *p* < .001 on SNR; with a significant condition-region interaction effect, *F*(4,1582) = 2.5, *p* = .04. Pairwise comparisons revealed that the occipital region had higher SNR during JA vs JW, *p* = .02 (Appendix D, Table A7, A8, A9**;** Fig. 6). A cluster-based permutation test on the channel level yielded a non-significant difference between conditions (higher SNR during JA vs JW) for the occipital channel POz and parietal channel Pz, cluster-level statistic = 4.34, *p* = .07; and a non-significant difference between conditions (higher SNR during JW vs JA) for the temporal channel T7, cluster-level statistic = −2.54, *p* = .14.

**Fig. 7:**
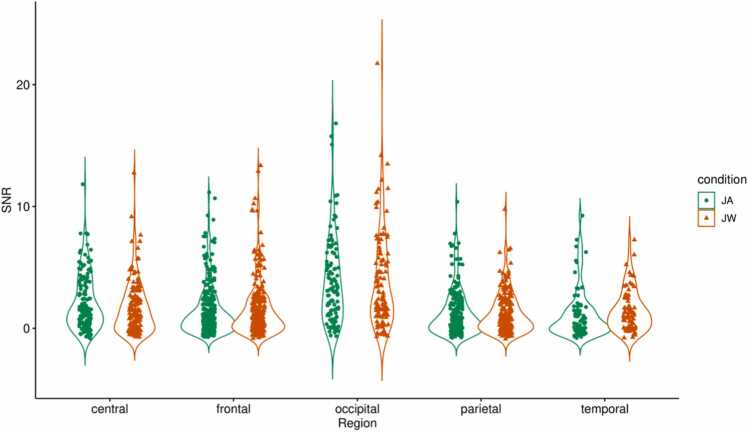
**LMM results - Mother SSVEP SNR (4 Hz) condition differences per scalp region.** SSVEP SNRs at 4 Hz were compared between conditions JA (in green) and JW (in orange) for mothers at central, frontal, occipital, parietal, and temporal regions during the whole duration of image presentation (0–2000 ms). SSVEP SNRs were not significantly higher during JA vs JW in any region.

For mothers, SSVEP SNRs for the same time window (0–2000 ms) averaged across all channels were also not significantly different between conditions, *t*(36) = 0.25, *p* = .81 (uncorrected). As an exploratory analysis step, based on visual inspection of the condition-specific topographic maps and their difference (Fig. 4**B**), a set of central, frontal, and parietal channels were also selected (C3, C4, CPz, Cz, AFz, F3, F4, Fz, P3, P4, Pz) for comparison (marked with a dashed line on Fig. 4**B**, in the following referred to as ‘central-frontal-parietal channels’). SNRs averaged across these channels were not significantly different between JA vs JW, *t*(36) = 1.15, *p* = .26 (uncorrected). However, when including only the central channels in the same test (C3, C4, CPz, Cz), SNRs averaged across these channels were significantly higher during JA vs JW, *t*(36) = 2.14, *p* = .04 (uncorrected, Fig. 5**B**). In the LMM, when SSVEP SNRs were compared between conditions and scalp-region-specific channels of interest, there was no significant overall effect of condition, *F*(1,1582) = 0.1, *p* = .75, but a significant effect of region, *F*(4,1582) = 81.12, *p* < .001 on SNR; with no significant condition-region interaction effect, *F*(4,1582) = 1.57, *p* = .18. None of the pre-defined contrasts (region-specific condition differences) were significant (Appendix D, Table A10, A11, A12**;** Fig. 7). A cluster-based permutation test on the channel level yielded a non-significant difference between conditions (higher SNR during JW vs JA) for the frontal channel F7, cluster-level statistic = −2.52, *p* = .13.

#### 3.2.2 SSVEP time course

**Fig. 8:**
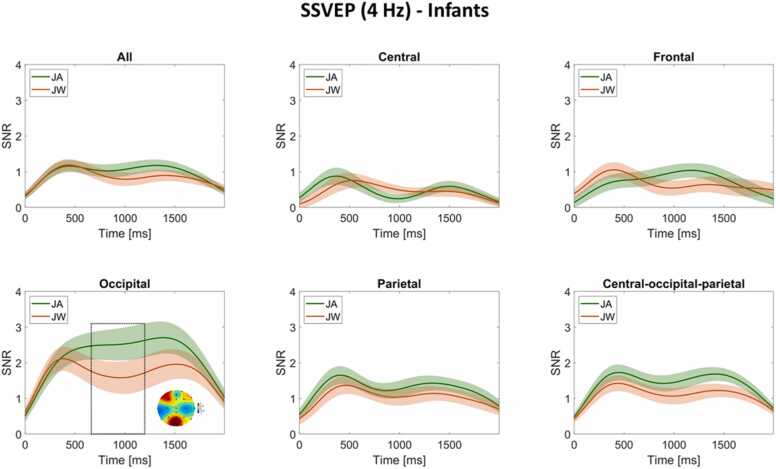
**SSVEP (4 Hz) time course for infants.** The time course of infant SSVEP SNR changes at 4 Hz averaged across all channels and scalp-region specific channels separately (i.e., central, frontal, occipital, parietal, and central-occipital-parietal channels) over the course of image presentation (0–2000 ms) during JA (green line) and JW (orange line). The time window with significant condition difference is marked with a black dashed rectangle (662–1198 ms for SNRs averaged across occipital channels). The topographic map during this time window depicts JA-JW condition differences in SNR. Shaded areas around each JA and JW line plot (depicting mean SSVEP SNR per condition) represent the standard error ( ± SE).

**Fig. 9:**
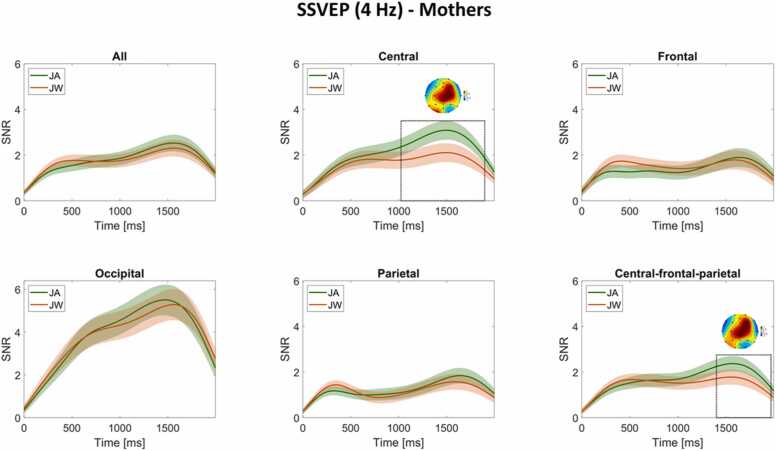
**SSVEP (4 Hz) time course for mothers.** The time course of mother SSVEP SNR changes at 4 Hz averaged across all channels and scalp-region specific channels separately (i.e., central, frontal, occipital, parietal, and central-frontal-parietal channels) over the course of image presentation (0–2000 ms) during JA (green line) and JW (orange line). Time windows with significant condition differences are marked with black dashed rectangles (1024–1898 ms and 1402–1970 ms in case of SNRs averaged across central, and central-frontal-parietal channels, respectively). Topographic maps during these time windows depict JA-JW condition differences in SNR. Shaded areas around each JA and JW line plot (depicting mean SSVEP SNR per condition) represent the standard error ( ± SE).

Next, we visualized the time course of infants’ and mothers’ SSVEP SNR changes over the whole image presentation time window (0–2000 ms), averaged across all channels and across scalp-region-specific channels of interest (central, frontal, occipital, parietal, and for infants only, central-occipital-parietal, while for mothers only, central-frontal-parietal) separately. Based on the results of the cluster-based permutation tests on the time dimension, we marked time points with SSVEP SNR condition differences (lower than *p* = .05) on SNR timeline plots and plotted their time-matched topographic maps of condition difference (Fig. 8, Fig. 9**)**.

For infants, a time window between 662 and 1198 ms showed a condition difference (higher SNR during JA vs JW) at occipital channels, cluster-level statistic = 599.27, *p* = .05 (Fig. 8). There were no other time windows with significant condition differences in other scalp regions. A cluster-based permutation test on all channels and time points found no significant condition differences (higher SNRs in JA vs JW or in JW vs JA) for infants, with all *p* values > .05 (Appendix E).

For mothers, two time windows pointed to a condition difference: one between 1024 and 1898 ms at central (cluster-level statistic = 1067.65, *p* = .01), and one between 1402 and 1970 ms at central-frontal-parietal channels (cluster-level statistic = 691.79, *p* = .05), both with higher SNRs during JA vs JW (Fig. 9). There were no other time windows with significant condition differences in other scalp regions. A cluster-based permutation test on all channels and time points found significant condition differences (higher SNRs in JA vs JW) for mothers at the following time windows and channels: 1080–1836 ms, CPz; 1162–1420 ms, C4; 1348–1968 ms, F4; 1380–1884 ms, Cz (cluster-level statistic = 2533, *p* = .045). There were non-significantly higher SNRs in JW vs JA for mothers at some other time windows and channels reported in Appendix E, with all *p* values > .05.

### 3.3 The effect of communicative signals on neural synchrony: AEC findings

First, AECs during mutual epochs were computed for each epoch individually within-dyad and within-condition on data averaged across all (except mastoid) channels, *z*-transformed and averaged across epochs. This resulted in one AEC value per dyad per condition. AECs were normally distributed (*p* = .17) as confirmed by Shapiro-Wilk test. Comparing AECs between conditions did not reveal a significant difference between JA and JW, *t*(30)= −0.77, *p* = .45. Next, the same analysis was performed for scalp-region-specific channels. AECs were normally distributed in all regions (all: *p* > .05) according to Shapiro-Wilk tests. AECs showed no difference between conditions for central, *t*(30) = 0.86, *p* = .40, frontal, *t*(30) = −0.65, *p* = .52, occipital, *t*(30) = 0.78, *p* = .44, and parietal channels, *t*(30) = −0.93, *p* = .36. Finally, we also compared AECs between infants’ central-parietal-occipital channels and mothers’ central channels, which showed condition differences in the within-subject analyses (see section 3.2.1., ‘SSVEP topographies’), but there was no significant difference between JA and JW, *t*(30) = 1.41, *p* = .17. We used the inverse Fisher transformation to obtain the mean Pearson correlation coefficients per condition, their standard deviation, and range (Table 1). As an exploratory analysis step, we compared epoch-level AECs between JA vs JW averaged across all channels, and occipital channels (that showed the highest SSVEP SNRs during mutual epochs, see Appendix C, Fig. A3). LMMs with condition as a fixed effect and dyad as a random effect did not reveal an effect of condition on AECs in case of all or occipital channels (Table 1, Table 2, Table 3, Table 4, Table 5**)**. We also compared channel-by-channel AECs between JA vs JW using a cluster-based permutation test but found no significant differences between conditions based on all channel-pairs (all adjusted *p* values >.05).

**Table 1:** Results of AEC Analyses

Regions	JA		JW		*t* (30)	*p*
	*M* (*SD*)	Range	*M* (*SD*)	Range		
All	-0.01 (0.18)	-0.5–0.36	0.02 (0.16)	-0.25–0.49	-0.77	0.45
Central	0.06 (0.14)	-0.21–0.37	0.02 (0.18)	-0.29–0.53	0.86	0.4
Frontal	-0.01 (0.14)	-0.28–0.28	0.01 (0.16)	-0.41–0.33	-0.65	0.52
Occipital	0.03 (0.23)	-0.54–0.54	-0.01 (0.18)	-0.41–0.43	0.78	0.44
Parietal	-0.03 (0.2)	-0.59–0.27	0.004 (0.19)	-0.25–0.5	-0.93	0.35
Central (mother) vs central-occipital-parietal (infant)	0.05 (0.22)	-0.4–0.48	-0.01 (0.19)	-0.41–0.38	1.41	0.17

*Note*. Mean, standard deviation and range of AECs averaged across channels (for region) and epochs per condition (JA, JW) for *n* = 30 infants. *T* and *p* values of dependent *t-*tests comparing region-specific AEC data between conditions (JA vs JW) are shown. All AEC values were Fisher *z*-transformed and then inverse Fisher *z*-transformed.

**Table 2:** Results of LMM for epoch-level AECs at all channels

**Fixed Effects Estimates**					
Term	Estimate	*SE*	*df*	*t*	*p*
Intercept	0.008	0.02	798	0.38	0.71
Condition (1)	-0.006	0.02	798	-0.26	0.8

*Note*. Fixed effects estimates of the LMM with dependent variable epoch-level AECs averaged across all channels, with condition (JA vs JW) as fixed effect and individual dyad as random effect. AEC values were Fisher *z*-transformed before fitting the model.

**Table 3:** Results of LMM for epoch-level AECs at all channels

**Estimated Marginal Means**					
				95% CI	
Condition	Estimate		*SE*	Lower	Upper
JA	0.003		0.03	-0.06	0.07
JW	0.014		0.03	-0.05	0.07

*Note*. Estimated marginal means of the LMM with dependent variable epoch-level AECs averaged across all channels, with condition (JA vs JW) as fixed effect and individual dyad as random effect. AEC values were Fisher *z*-transformed before fitting the model.

**Table 4:** Results of LMM for epoch-level AECs at occipital channels

**Fixed Effects Estimates**							
Term	Estimate	*SE*		*df*	*t*		*p*
Intercept	0.004	0.03		27.58	0.15		0.89
Condition (1)	0.025	0.02		200.42	1.03		0.31

*Note*. Fixed effects estimates of LMM with dependent variable epoch-level AECs averaged across occipital channels (O1, O2, POz), with condition (JA vs JW) as fixed effect and individual dyad as random effect. AEC values were Fisher *z*-transformed before fitting the model.

**Table 5:** Results of LMM for epoch-level AECs at occipital channels

**Estimated Marginal Means**				
			95% CI	
Condition	Estimate	*SE*	Lower	Upper
JA	0.03	0.04	-0.05	0.11
JW	-0.02	0.03	-0.09	0.05

*Note*. Estimated marginal means of LMM with dependent variable epoch-level AECs averaged across occipital channels (O1, O2, POz), with condition (JA vs JW) as fixed effect and individual dyad as random effect. AEC values were Fisher *z*-transformed before fitting the model.

## 4 Discussion

In the current study, we investigated the effects of communicative signals, namely eye contact, IDS and pointing during joint attention on 11–12-month-old infants’ and their mothers’ brain activity in a naturalistic, social interactional context applying RVS combined with EEG hyperscanning. We tested if communicative cues could enhance mother-infant dyads’ attention, tracked individually by SSVEP, the evoked neural responses elicited by RVS. This was achieved by presenting flickering visual images of objects and animals on a computer screen that dyads attended to in a joint attention interaction with or without maternal communicative cues. In accordance with our hypothesis, we found that communicative cues enhanced both infants’ and mothers’ attention (evidenced by increased individual SSVEPs) during joint attention. In addition, we assessed if infants and mothers aligned their attention more due to communicative signals and showed more similar neural activity (i.e., neural synchrony) during joint attention with communicative engagement. We expected that neural synchrony would be reflected in increased SSVEP amplitude envelope correlations between the dyads’ brain activity. Interestingly, we did not find evidence for an effect of communicative signals on neural synchrony during joint attention.

Infants showed increased visual processing of objects during joint attention with vs without communicative cues. This was indicated by higher neural responses at infants’ central, parietal and occipital EEG electrode sites during the whole duration of joint attention to the presented images. This is in line with prior EEG studies that found an increase in 5- and 9-month-old infants’ neural responses (ERPs) to objects following a brief phase of eye contact with an adult, particularly in fronto-central locations (Parise et al., 2008, Striano et al., 2006b), likely indexing enhanced object processing. During joint attention to objects, event-related desynchronization in the alpha frequency band has also been observed in 6- and 9-month-old infants at central, frontal and parietal EEG channels (Hoehl et al., 2014, Rayson et al., 2019). Even though we did not perform source localization for our EEG data, based on the topographic distribution of infants’ SSVEP responses, we found a broader neural activation over central, frontal, occipital and parietal areas during JA vs JW. This result is supported by studies showing that SSVEPs do not only reflect perceptual aspects of incoming visual information (primarily seen over the occipital cortex) but are also involved in higher-level sensory processes, including categorization (e.g., Farzin et al., 2012), or the formation of object representations (Radtke et al., 2021). In the meantime, infants showed an increase in neural responses specific to the occipital electrode sites at the beginning of joint attention (approximately 600–1200 ms) with communicative engagement. This likely indicates enhanced visual processing due to an initial attention boost and is in line with prior work that found a larger Nc ERP component during joint attention in this time window (Parise et al., 2008; Striano et al., 2006b). Our findings support the view that communicative cues during social interactions have a specific importance for the infant brain (Grossmann et al., 2008, Michel et al., 2019), by facilitating attention processes in a social context (Niedźwiecka et al., 2018, Wass et al., 2020b), and promoting higher-level object processing on a neural level from early on in infancy (Wahl et al., 2019).

The results add to findings from previous research that applied RVS in a non-social context and pinpointed the generic enhancing effect of attention on SSVEP responses. As early as the age of 3 months (Robertson et al., 2012), infants’ neural responses increased to a flickering, rotating toy vs a flickering, steady toy. Four-month-olds’ SSVEP amplitudes were modulated by overt and covert attention, and following habituation, showed recovery upon presentation to a novel stimulus (Christodoulou et al., 2018). Nine-month-old infants’ SSVEPs were found to increase in response to unexpected vs expected events, potentially facilitating the integration of novel information into existing representations (Köster et al., 2019). Here we show that the social modulation of infants' attention and visual processing via communicative cues can likewise be tracked by measuring SSVEP responses in social contexts.

In our study, the two conditions were carefully matched for infants’ attention levels by including a non-social, attention-enhancing video before every third image in the JW trials, while asking mothers to communicatively engage with the infants at the same rate, before every third image in the JA trials. Since our two conditions were matched for attention levels as much as possible, the observed effect of increased neural activity of infants during JA can be more specifically attributed to communicative cues rather than to a generic attention boost. Yet, this possibility cannot be fully outruled as mothers were directed to keep their index finger on the screen, pointing out the objects throughout the JA image presentation. Incorporating an additional, non-social, but attention-enhancing condition to the study (i.e., a pointer to the screen such as a pointer stick) would have helped to disentangle the more fine-grained effects of non-social and social attention (with or without communicative cues) on infants’ SSVEP responses. Furthermore, infants’ behaviour, specifically gaze duration to the images was significantly increased by communicative cues in JA compared to non-social attention cues in JW. This is in accordance with prior studies highlighting the unique role of communicative signals in infants’ attention and learning processes (Okumura et al., 2020, Wass et al., 2020b). Regarding EEG data quality, this finding also constituted a limitation for the study, as infants had a higher number of clean EEG epochs in the JA than in the JW condition.

Mothers, like their infants, showed increased neural responses (higher SSVEPs) to images during joint attention with vs without communicative cues. This effect was localized at central EEG electrode sites over the whole duration (but especially druing the 1000-2000 ms time window) of the joint attention interaction. In addition, mothers had higher SSVEPs over central-frontal-parietal channels specifically from the second half (approximately 1400–2000 ms) of the JA interaction with communicative cues. Mothers’ attention and subsequent visual object processing was thus similarly facilitated by communicative cues during joint attention as their infants’. Since the topographic distribution of condition difference in brain activity was primarily present at central channels, we can conclude that mothers also displayed a higher-level of object processing beyond low-level sensory information usually processed at occipital areas (Radtke et al., 2021). Moreover, while infants’ SSVEPs increased from image onset due to social cues, mothers displayed a more extensive topography of the increased neural response (from central to frontal and parietal channels) with a slight delay, suggesting a sort of temporal tracking of infants’ attention dynamics.

The findings of increased maternal neural responses during joint attention with communicative cues highlight the relevance of social factors in modulating attentional processes. Thus, our study extends previous results on the enhancing effect of non-social attention on adults’ SSVEPs (Gulbinaite et al., 2019, Müller et al., 1998) by showing that social cues have similar effects on this visual response. As in the case of infants, since the two conditions only differed in the presence or absence of communicative cues, we conclude that social engagement with their infant could facilitate mothers’ object processing and lead to increased attention. In contrast with infants, mothers’ gaze duration to the images was not affected by communicative cues in JA compared to non-social attention cues in JW. However, mothers were instructed to communicatively engage with their infants in a relatively structured manner in the JA condition to facilitate EEG data recording. This posed a further limitation for the study: mothers likely did not behave as naturally over the course of the task as they would have otherwise, e.g., in a free-flowing social interaction. During JA, they were instructed to actively engage with their infant, thus mothers' higher attention levels during JA vs JW could be partially attributed to higher task demands besides communicative cues. Due to movement and talking, they also had a lower number of clean EEG epochs during the JA compared to the JW condition and lower SSVEP SNR in the first epochs of JA vs JW. This is a constraint that future studies could address by using additional control conditions (e.g., caregiver talking to another adult).

Our neural findings on the individual level thus revealed that communicative cues lead to increased attention and visual processing of information in the dyad. Intriguingly, our hypothesis that communicative cues will contribute to a dynamic, mutual adjustment between infants’ and caregivers’ neural activity and result in enhanced neural synchrony was not confirmed. We found no differences in neural synchrony between infants and caregivers during joint attention with or without communicative cues. Even though parents have been found to be neurally responsive to their infants and infants’ attention is facilitated by this neural responsiveness, e.g., during social play (Wass et al., 2018b), recent EEG hyperscanning studies also could not identify a link between communicative cues such as mutual gaze and neural synchrony in complex, naturalistic contexts (e.g., Marriott Haresign et al., 2023). In the case of mutual gaze, this could be due to a more simple, intra-brain processing of eye contact episodes, that did not affect dynamic neural processes, or due to data quality concerns such as the presence of artifacts. Meanwhile, other EEG hyperscanning studies revealed mother-infant biobehavioral synchrony in early interactions, but not with a particular focus on disentangling the effects of communicative cues on shared neural activity (e.g., Endevelt-Shapira and Feldman, 2023).

The absence of an effect of communicative cues on neural synchrony in our study may have several reasons. First, communicative cues during joint attention may only serve to realign the attention of infants and caregivers but do not lead to more neural synchrony than sharing attentional focus without communicative cues. Second, infants’ attention increases (higher SSVEP SNRs) were mostly localized to central, occipital, and parietal electrode sites, while another topography of the response was observed for mothers (i.e., central, frontal, and parietal). The time course of attention enhancement was also different in the dyad: infants showed higher neural responses earlier than mothers during JA. These results suggest that the alignment of attention in the dyad does not necessarily unfold simultaneously between the infant and caregiver and might have complicated the detection of neural synchrony increases over the course of mutually attended epochs in JA vs JW. In fact, there is a recent theoretical account on flexible, multimodal synchrony that emphasizes the importance of context and individual differences for the emergence of synchrony (Gordon et al., 2023). In certain contexts, such as social coordination and social learning, it may prove more adaptive for infant and caregiver to fluctuate between synchronous and non-synchronous episodes, giving rise to flexible and dynamic synchrony patterns in visual attention that might not be captured by the neural synchrony measures assessed here. Third, a recent study with 10–12-month-olds unveiled that infants actually display few ostensive cues before engaging in joint attention episodes with their caregiver and do not increase their neural activity (Phillips et al., 2023). These findings suggest that infants at this age may be less proactive in initiating and maintaining joint attention than previously thought, which can have implications for neural synchrony during joint attention with communicative cues. Potentially, a task requiring more explicit information transfer from mother to infant would have evoked higher levels of neural synchronisation (see e.g., Pan et al., 2020, for an example learning task in adult dyads). Lastly, we cannot rule out that our preregistered analysis could not fully capture the complexity of neural synchrony patterns in our data that differed from neural synchrony assessment in free-flowing interactional hyperscanning studies. Additional limitations could arise from the fact that our epochs for extracting AEs were rather short (2 s), while AECs become more sensitive with longer-range dependencies (Bruns et al., 2000, Zamm et al., 2021). This could be addressed by future RVS paradigms with infants using longer trials. Further, AECs might capture neural synchrony better in naturalistic interactions, when brain activity has more dynamic oscillatory changes than in the case of perceiving periodic rhythmic stimuli (e.g., Zamm et al., 2023).

We applied RVS as a novel approach to quantify attention and neural synchrony in a social interaction context. Our results demonstrate that RVS is a promising tool to assess neural dynamics in infant-caregiver dyads and track dynamic mutual adjustment in dyads’ attention. A limitation of this method is that we still know relatively little about the interactions and interdependencies between neural responses elicited by RVS and endogenous oscillations (Bánki et al., 2022, Köster et al., 2023b, Wass et al., 2022). The findings reported here provide an interesting avenue for future research to explore this methodology in the study of neural dynamics during early social interactions. Future studies could build on our results to investigate other social-cognitive processes such as early learning, perspective-taking, or the formation of representations in interactive contexts.

## 5 Conclusion

Communicative cues are essential building blocks of early social interactions such as joint attention. Yet relatively little is known about how communicative signals affect the neural dynamics of infants and caregivers in dynamic social exchanges fundamental to social development and learning. Measuring infant-caregiver dyads’ brain activity in naturalistic interactions remains challenging (Turk et al., 2022, Wass and Goupil, 2022), but recent findings attested to infants’ neural sensitivity to mutual gaze (Piazza et al., 2020) and their attention being followed during joint attention (Phillips et al., 2023). Caregivers’ neural activity was also found to be predictive of changes in infants’ attention (Wass et al., 2018b). Results from the current study provide further evidence that maternal communicative cues lead to enhanced neural responses to visual stimuli in infant-caregiver dyads, pointing to increased attention in early interactions accompanied with communicative cues. This finding highlights the essential role of communicative cues in facilitating information processing during social learning in infancy. As communicative cues did not modulate neural synchrony in the current study, future research is needed to elucidate the fine-grained aspects of communicative engagement that give rise to neural synchrony in naturalistic contexts. In all, our study sheds light on how communicative cues modulate neural responses to objects in joint attention and thereby shape shared visual experiences of infants and caregivers.

## CRediT Author Statement

**Anna Bánki:** Conceptualization, Methodology, Software, Formal analysis, Investigation, Data curation, Writing – original draft, Writing – review & editing, Visualization, Project administration. **Moritz Köster:** Conceptualization, Methodology, Software, Writing – review & editing, Supervision. **Radoslaw Martin Cichy:** Conceptualization, Methodology, Writing – review & editing, Supervision. **Stefanie Hoehl:** Conceptualization, Methodology, Resources, Writing – review & editing, Supervision, Project administration, Funding acquisition.

## Funding

Funding for this research, including open access publication fees, was provided by the University of Vienna. M.K. and S.H. were supported by the 10.13039/100004807DFG and FWF jointly (grant numbers: KO 6028/1-1; I 4332-B). R.M.C. was supported by the DFG (CI 241/1-1; CI 241/3-1; CI 241/1-7) and the 10.13039/501100000781European Research Council (ERC-StG-2018-803370).

## Declaration of Competing Interest

The authors declare that they have no known competing financial interests or personal relationships that could have appeared to influence the work reported in this paper.

## Figures and Tables

**Table A1:** Results of LMM for region-specific SSVEP SNRs for infants in first epochs

**Fixed Effects Estimates**					
Term	Estimate	*SE*	*df*	*t*	*p*
Intercept	0.31	0.12	161	2.63	< .01
Condition (JW)	0.1	0.13	1582	0.78	0.43
Frontal	0.06	0.11	1582	0.57	0.57
Occipital	1.22	0.14	1582	8.94	< .001
Parietal	0.48	0.12	1582	4.03	< .001
Temporal	0.05	0.15	1582	0.31	0.76
Condition (JW) * Frontal	-0.04	0.15	1582	-0.24	0.81
Condition (JW) * Occipital	-0.31	0.19	1582	-1.6	0.11
Condition (JW) * Parietal	-0.15	0.17	1582	-0.86	0.39
Condition (JW) * Temporal	-0.04	0.22	1582	-0.17	0.87

*Note*. Fixed effects estimates of LMM with dependent variable SSVEP SNR, with condition (JA vs JW) and scalp-regions as fixed effects, and individual as random effect.

**Table A2:** Results of LMM for region-specific SSVEP SNRs for infants in first epochs

**Estimated Marginal Means**						
					95% CI	
Condition	Region	Estimate		*SE*	Lower	Upper
JA	Central	0.31		0.12	0.08	0.54
JW	Central	0.4		0.12	0.17	0.63
JA	Frontal	0.37		0.1	0.17	0.56
JW	Frontal	0.43		0.1	0.23	0.62
JA	Occipital	1.52		0.13	1.27	1.77
JW	Occipital	1.31		0.13	1.06	1.56
JA	Parietal	0.79		0.11	0.57	1
JW	Parietal	0.74		0.11	0.52	0.96
JA	Temporal	0.35		0.15	0.06	0.64
JW	Temporal	0.41		0.15	0.13	0.7

*Note*. Estimated marginal means of LMM with dependent variable SSVEP SNR, with condition (JA vs JW) and scalp-regions as fixed effects, and individual as random effect.

**Table A3:** Results of LMM for region-specific SSVEP SNRs for infants in first epochs

**Contrasts**					
Contrast	Region	Estimate	*SE*	*t*	*p*
JA-JW	Central	-0.1	0.13	-0.78	0.94
JA-JW	Frontal	-0.06	0.09	-0.69	0.97
JA-JW	Occipital	0.21	0.15	1.44	0.55
JA-JW	Parietal	0.05	0.11	0.42	0.99
JA-JW	Temporal	-0.06	0.18	-0.35	0.99

*Note*. Contrasts for multiple comparisons, *p* values adjusted by using multivariate *t*-distribution.

**Table A4:** Results of LMM for region-specific SSVEP SNRs for mothers in first epochs

**Fixed Effects Estimates**							
Term	Estimate	*SE*		*df*	*t*		*p*
Intercept	0.52	0.18		87.67	2.92		< .001
Condition (JW)	0.45	0.18		1195	2.53		0.01
Frontal	-0.4	0.15		1195	-2.64		< .01
Occipital	1.15	0.19		1195	6.02		< .001
Parietal	-0.25	0.17		1195	-1.51		0.13
Temporal	-0.25	0.22		1195	-1.17		0.24
Condition (JW) * Frontal	0.17	0.22		1195	0.76		0.45
Condition (JW) * Occipital	0.6	0.27		1195	2.21		0.03
Condition (JW) * Parietal	0.1	0.24		1195	0.42		0.67
Condition (JW) * Temporal	0.06	0.31		1195	0.19		0.85

*Note*. Fixed effects estimates of LMM with dependent variable SSVEP SNR, with condition (JA vs JW) and scalp-regions as fixed effects, and individual as random effect.

**Table A5:** Results of LMM for region-specific SSVEP SNRs for mothers in first epochs

**Estimated Marginal Means**					
				95% CI	
Condition	Region	Estimate	*SE*	Lower	Upper
JA	Central	0.52	0.18	0.17	0.87
JW	Central	0.97	0.18	0.61	1.32
JA	Frontal	0.12	0.15	-0.2	0.43
JW	Frontal	0.73	0.15	0.42	1.04
JA	Occipital	1.67	0.19	1.29	2.05
JW	Occipital	2.71	0.19	2.33	3.09
JA	Parietal	0.27	0.17	-0.07	0.6
JW	Parietal	0.81	0.17	0.48	1.15
JA	Temporal	0.27	0.22	-0.16	0.7
JW	Temporal	0.77	0.22	0.34	1.2

*Note*. Estimated marginal means of LMM with dependent variable SSVEP SNR, with condition (JA vs JW) and scalp-regions as fixed effects, and individual as random effect.

**Table A6:** Results of LMM for region-specific SSVEP SNRs for mothers in first epochs

**Contrasts**					
Contrast	Region	Estimate	*SE*	*t*	*p*
JA-JW	Central	-0.45	0.18	-2.53	**0.06**
JA-JW	Frontal	-0.61	0.13	-4.9	**< .0001**
JA-JW	Occipital	-1.04	0.2	-5.11	**< .0001**
JA-JW	Parietal	-0.55	0.16	-3.46	**< .01**
JA-JW	Temporal	-0.5	0.25	-2.02	0.2

*Note*. Contrasts for multiple comparisons, *p* values adjusted by using multivariate *t*-distribution. Significant *p* values are highlighted in bold.

**Table A7:** Results of LMM for region-specific SSVEP SNRs for infants

**Fixed Effects Estimates**					
Term	Estimate	*SE*	*df*	*t*	*p*
Intercept	0.49	0.14	146.78	3.53	< .001
Condition (JW)	-0.03	0.15	1582	-0.2	0.84
Frontal	0.23	0.13	1582	1.76	0.08
Occipital	1.69	0.16	1582	10.59	< .001
Parietal	0.79	0.14	1582	5.65	< .001
Temporal	-0.12	0.18	1582	-0.68	0.5
Condition (JW) * Frontal	-0.02	0.18	1582	-0.09	0.93
Condition (JW) * Occipital	-0.46	0.23	1582	-2.04	0.04
Condition (JW) * Parietal	-0.21	0.2	1582	-1.06	0.29
Condition (JW) * Temporal	0.31	0.26	1582	1.19	0.23

*Note*. Fixed effects estimates of LMM with dependent variable SSVEP SNR, with condition (JA vs JW) and scalp-regions as fixed effects, and individual as random effect.

**Table A8:** Results of LMM for region-specific SSVEP SNRs for infants

**Estimated Marginal Means**					
				95% CI	
Condition	Region	Estimate	*SE*	Lower	Upper
JA	Central	0.49	0.14	0.22	0.77
JW	Central	0.46	0.14	0.19	0.74
JA	Frontal	0.72	0.12	0.48	0.96
JW	Frontal	0.67	0.12	0.44	0.91
JA	Occipital	2.19	0.15	1.89	2.49
JW	Occipital	1.69	0.15	1.39	1.99
JA	Parietal	1.29	0.13	1.03	1.55
JW	Parietal	1.05	0.13	0.79	1.31
JA	Temporal	0.37	0.17	0.03	0.71
JW	Temporal	0.65	0.17	0.3	0.99

*Note*. Estimated marginal means of LMM with dependent variable SSVEP SNR, with condition (JA vs JW) and scalp-regions as fixed effects, and individual as random effect.

**Table A9:** Results of LMM for region-specific SSVEP SNRs for infants

**Contrasts**					
Contrast	Region	Estimate	*SE*	*t*	*p*
JA-JW	Central	0.03	0.15	0.2	0.99
JA-JW	Frontal	0.05	0.11	0.44	0.99
JA-JW	Occipital	0.49	0.17	2.88	**0.02**
JA-JW	Parietal	0.24	0.13	1.82	0.3
JA-JW	Temporal	-0.28	0.21	-1.32	0.65

*Note*. Contrasts for multiple comparisons, *p* values adjusted by using multivariate *t*-distribution. Significant *p* values are highlighted in bold.

**Table A10:** Results of LMM for region-specific SSVEP SNRs for mothers

**Fixed Effects Estimates**					
Term	Estimate	*SE*	*df*	*t*	*p*
Intercept	2.11	0.28	69.2	7.6	< .001
Condition (JW)	-0.49	0.22	1582	-2.24	0.03
Frontal	-0.73	0.19	1582	-3.85	< .001
Occipital	1.70	0.24	1582	7.25	< .001
Parietal	-0.86	0.21	1582	-4.18	< .001
Temporal	-0.97	0.27	1582	-3.62	< .001
Condition (JW) * Frontal	0.59	0.27	1582	2.21	0.03
Condition (JW) * Occipital	0.47	0.33	1582	1.42	0.16
Condition (JW) * Parietal	0.43	0.29	1582	1.47	0.14
Condition (JW) * Temporal	0.78	0.38	1582	2.08	0.04

*Note*. Fixed effects estimates of LMM with dependent variable SSVEP SNR, with condition (JA vs JW) and scalp-regions as fixed effects, and individual as random effect.

**Table A11:** Results of LMM for region-specific SSVEP SNRs for mothers

**Estimated Marginal Means**					
				95% CI	
Condition	Region	Estimate	*SE*	Lower	Upper
JA	Central	2.11	0.28	1.55	2.66
JW	Central	1.62	0.28	1.07	2.17
JA	Frontal	1.38	0.26	0.87	1.89
JW	Frontal	1.48	0.26	0.97	1.99
JA	Occipital	3.81	0.29	3.23	4.39
JW	Occipital	3.79	0.29	3.22	4.37
JA	Parietal	1.24	0.27	0.71	1.78
JW	Parietal	1.18	0.27	0.65	1.72
JA	Temporal	1.14	0.32	0.51	1.77
JW	Temporal	1.44	0.32	0.81	2.06

*Note*. Estimated marginal means of LMM with dependent variable SSVEP SNR, with condition (JA vs JW) and scalp-regions as fixed effects, and individual as random effect.

**Table A12:** Results of LMM for region-specific SSVEP SNRs for mothers

**Contrasts**					
Contrast	Region	Estimate	*SE*	*t*	*p*
JA-JW	Central	0.49	0.22	2.24	0.12
JA-JW	Frontal	-0.1	0.15	-0.66	0.97
JA-JW	Occipital	0.01	0.25	0.06	1
JA-JW	Parietal	0.06	0.2	0.31	0.99
JA-JW	Temporal	-0.3	0.231	-0.96	0.87

*Note*. Contrasts for multiple comparisons, *p* values adjusted by using multivariate *t*-distribution.

## Data Availability

All pre-processed data will be made available upon request. Video data will remain confidential and will not be shared due to privacy reasons of participants, including minors.

## Appendix B – Supplementary Analysis: Intra-brain analysis 1.

As a further exploratory analysis step, we compared only the first epochs in each trial between conditions, since the first JA epochs were accompanied with all three communicative cues. For this analysis, mothers who did not have at least one first epoch per condition were excluded (*n* = 9).^3^ All infants (*n* = 37) had at least one first epoch per condition. Infants had good quality data for 13.24% of all epochs that were first epochs in JA (*SD* = 6.29%, range: 2–30%), and 11% in the case of JW (*SD* = 4.09%, range: 3–20%). They had significantly more first epochs retained in the JA vs the JW condition, *t*(36) = 2.18, *p* = .03. Mothers had suitable data quality for 10% of all epochs that were first epochs in JA (*SD* = 5.61%, range: 2–22%), and 23% in the case of JW (*SD* = 6.67%, range: 2–32%). Mothers retained significantly more first epochs in the JW vs the JA condition, *t*(27) = −9.84, *p* < .001.

We first performed an EEG data quality check identical to the one for our main analysis: we statistically tested if SSVEP SNRs differed from noise level during the subset of first attended epochs, separately for infants and mothers. We compared the condition-level grand mean SNR values (averaged across participants included in the first-epoch analysis, all first epochs per participant, and all electrodes) against the noise level of 0 at 4 Hz. Grand mean SNR values averaged across all electrodes and the whole image presentation duration (0–2000 ms) were significantly different from noise level during JA for infants, *t*(36) = 6.48, *p* < .001, and mothers, *t*(27) = 3, *p* < .01, as well as during JW for infants, *t*(36) = 6.28, *p* < .001, and mothers, *t*(27) = 6.06, *p* < .001 (Fig. A2).

For infants, SSVEP SNRs in the first epochs for the whole image presentation duration (0–2000 ms) averaged across all channels were not significantly different between conditions, *t*(36) = −0.06, *p* = .96 (uncorrected). A LMM identical to the one conducted for the main analyses did not find any condition effect, only a significant effect of region on SSVEP SNR. However, there was no significant difference in any pre-selected regions between conditions (Table A1, Table A2, Table A3). Cluster-based permutation tests on the time domain for separate regions showed no significant condition differences in SNRs at any of the electrode sites (all *p* values >.05). A cluster-based permutation test on the channel-time space also did not reveal any significant condition differences (all *p* values >.05).

For mothers, SSVEP SNRs in the first epochs averaged across all channels were significantly different between conditions, with lower SNRs in JA vs JW, *t*(27) = −3.12, *p* < .01 (uncorrected). A LMM identical to the one in the main analysis found that condition and region both had a significant effect on SSVEP SNRs: pairwise contrasts identified significantly lower SNRs during JA vs JW in central, frontal, occipital, and parietal regions (Table A4, Table A5, Table A6). Cluster-based permutation tests on the time domain for regions revealed significantly lower SNRs during JA vs JW at all electrode sites between 72 and 1918 ms, cluster-level statistic = −2428.03, *p* < .01; at frontal channels between 168 and 1846 ms, cluster-level statistic = −1778.42, *p* = .01; at parietal channels between 74 and 1008 ms, cluster-level statistic = −1433.08, *p* = .01, and 1100–1840 ms, cluster-level statistic = −840.24, *p* = .04; and central-frontal-parietal channels between 100 and 722 ms, cluster-level statistic = −915.43, *p* = .04, and 1226–1822 ms, cluster-level statistic = −689.46, *p* = .08. A cluster-based permutation test on the channel-time space also pointed to significant condition differences, specifically lower SNR in JA vs JW (1) between 202 and 272 ms at P3; and 202–416 ms at P4, POz, Pz, cluster- level statistic = −1120, *p* = .05; (2) between 1384 and 1694 ms at O2, POz, Pz, cluster-level statistic = −1279.5, *p* = .04; and a non-significant condition difference (lower SNR in JA vs JW) between 1206 and 1270 ms at P4, POz, Pz, cluster-level statistic = −216.95, *p* = .09.

## Appendix C – Supplementary Analyses: Inter-brain analysis

Before conducting the AEC analysis, we statistically evaluated if SSVEP amplitude SNRs differed from noise level during the subset of epochs that were mutually attended by infants and mothers. We compared the condition-level grand mean amplitude SNR values (averaged across participants included in the AEC analysis, across all mutually attended epochs per dyad, and all electrodes) against the noise level of 0 at 4 Hz..

## Appendix E – Supplementary Analysis: Intra-brain analysis 2.

We applied cluster-based permutation tests on the channel-time dimensions for all channels and time points to test for condition differences in SSVEP SNRs. For infants, there were non-significantly higher SSVEP SNRs during JA vs JW at times and channels: 0–2 ms, T7 (cluster-level statistic = 4.09, *p* = .90); 722–1382 ms, POz (cluster-level statistic = 768.04, *p* = .33); 800–1308 ms, F3, and 886–1320 ms, F7 (cluster-level statistic = 1111.8, *p* = .20); 1410–1484 ms, O1 (cluster-level statistic = 77.12, *p* = .90); 1488–1648 ms, CPz (cluster-level statistic = 169.85, *p* = .81). The time periods and channels with non-significantly higher SSVEP SNRs during JW vs JA were identified as 442–576 ms, T7 (cluster-level statistic = −139.38, *p* = .83); 1120–1434 ms, T7 (cluster-level statistic = −343.06, *p* = .67). For mothers, there were non-significantly higher SSVEP SNRs during JW vs JA at times and channels: 50–494 ms, P4 (cluster-level statistic = −591.89, *p* = .43); 1288–1604 ms, F7 (cluster-level statistic = −345.7, *p* = .63); 1990–2000 ms, O2 (cluster-level statistic = −12.25, *p* = .85).

## Footnotes

1 https://aspredicted.org/mw9qp.pdf

2 In this subsample, infants’ age was *M* = 12.2 in months (*SD* = 0.61, range: 11.13–13.47; 17 girls), and mothers’ age was *M* = 34.2 in years (*SD* = 5.3). The number of infants was balanced between the two age groups of 11 months 0–30 days (*n =* 14) and 12 months 0–30 days (*n =* 13) or older, and a small number of infants was between 13 months 0–14 days (*n =* 4). The number of dyads was balanced between condition order (48% started with JA and 52% with JW).
